# Mycobacteriophage Lysis Enzymes: Targeting the Mycobacterial Cell Envelope

**DOI:** 10.3390/v10080428

**Published:** 2018-08-14

**Authors:** Maria João Catalão, Madalena Pimentel

**Affiliations:** Research Institute for Medicines (iMed.ULisboa), Faculty of Pharmacy, Universidade de Lisboa, 1649-003 Lisboa, Portugal; mjcatalao@ff.ulisboa.pt

**Keywords:** mycobacteriophages, Ms6, endolysins, LysB, lysis, mycobacteria

## Abstract

Mycobacteriophages are viruses that specifically infect mycobacteria, which ultimately culminate in host cell death. Dedicated enzymes targeting the complex mycobacterial cell envelope arrangement have been identified in mycobacteriophage genomes, thus being potential candidates as antibacterial agents. These comprise lipolytic enzymes that target the mycolic acid-containing outer membrane and peptidoglycan hydrolases responsive to the atypical mycobacterial peptidoglycan layer. In the recent years, a remarkable progress has been made, particularly on the comprehension of the mechanisms of bacteriophage lysis proteins activity and regulation. Notwithstanding, information about mycobacteriophages lysis strategies is limited and is mainly represented by the studies performed with mycobacteriophage Ms6. Since mycobacteriophages target a specific group of bacteria, which include *Mycobacterium tuberculosis* responsible for one of the leading causes of death worldwide, exploitation of the use of these lytic enzymes demands a special attention, as they may be an alternative to tackle multidrug resistant tuberculosis. This review focuses on the current knowledge of the function of lysis proteins encoded by mycobacteriophages and their potential applications, which may contribute to increasing the effectiveness of antimycobacterial therapy.

## 1. Introduction

The growing problem of multidrug-resistant bacteria is currently considered a threat to global health [1,2]. This, together with a disinvestment of the pharmaceutical industry in the development of novel-acting antibiotics [3] and the urgent need of alternative therapeutics, has renewed the interest in the use of bacteriophages to fight bacterial infections. This is due to the bacteriolytic properties of phages, which lead to cell death. Double-stranded (ds) DNA phages end a lytic cycle by releasing the newly synthesized phage particles from the infected cells to the environment. To achieve this, they have to overcome each barrier of the cell envelope by producing lysis proteins that exert their action at an appropriate time. It is this capacity of lysing bacteria that is being explored to use whole phages or their derived proteins not only for therapeutic applications, but also for biocontrol in the food industry and in detection of pathogenic bacteria [4,5,6,7,8].

In dsDNA phages, lysis begins with the function of holins, which are membrane proteins that accumulate in the cytoplasmic membrane (CM) and that at a genetically determined time oligomerize and permeabilize the membrane forming holes. This allows endolysins, which are enzymes that cleave specific bonds in the peptidoglycan (PG), to access their substrate and disrupt the cell wall (CW) [9,10]. Since PG is the major component of the bacterial CW and its integrity is essential for cell viability, breaking of this structure leads to osmotic lysis and the bacterial cell dies [11]. For this reason, the use of purified endolysins against both Gram-positive and Gram-negative bacteria has been extensively studied aiming their application as antimicrobials in bacterial infections [12,13]. Since, with very few exceptions [14], endolysins are primarily ineffective against Gram-negative bacteria, engineered endolysins are being developed to circumvent the bacterial outer membrane (OM), which is a barrier to the external accessibility of natural endolysins to the PG [15].

For phages infecting Gram-negative hosts a third-class of lysis proteins has recently been shown to be essential for lysis [16,17]. Spanins are proteins that span the entire periplasm, linking the outer and inner bacterial membranes. After disruption of the PG by the endolysins, spanins undergo conformational changes that lead to the fusion of both membranes which results in removal of the OM and consequently in phage release [18,19].

In Gram-negative infecting phages, host lysis is a three step event mediated by holins, endolysins, and spanins in a sequential pathway, in which by this order each lysis proteins eliminates the cell envelope barriers to phage release, i.e., CM, PG, and OM respectively [17].

Although we have been witnessing an increased knowledge in the way bacteriophage lysis proteins exert their function, particularly from the studies performed with bacteriophage lambda [9,20], information about lysis induced by mycobacteriophages is scarce. At the time of this writing more than 9800 mycobacteriophages have been isolated, over 1615 were sequenced [21], and 350 complete genome sequences are available in GenBank. Based on nucleotide sequence similarity these phages were grouped in clusters (29 designated from A to AC) and the genes assorted into phamilies (Phams) according to the amino acid sequence similarity of their products [21,22,23]. Despite the huge number of isolated mycobacteriophages, studies on lysis systems and their associated proteins are restricted to a few phages and are mainly confined to the ones performed with mycobacteriophages Ms6 [24,25] and D29 [26,27,28,29]. Since mycobacteriophages infect a particular group of bacteria, which include the pathogenic *M. tuberculosis* responsible for one of the leading causes of death worldwide [30], characterization of the activity of these lysis proteins opens new avenues to explore them as novel tools to destroy mycobacteria cells.

This review summarizes the available information on the activity of lysis enzymes encoded by mycobacteriophages and their potential applications. For more detailed information on the mechanisms of mycobacteriophage-induced lysis there are other reviews that the reader may find useful [10,20,25].

## 2. The Mycobacteria Cell Envelope

**Figure 1:**
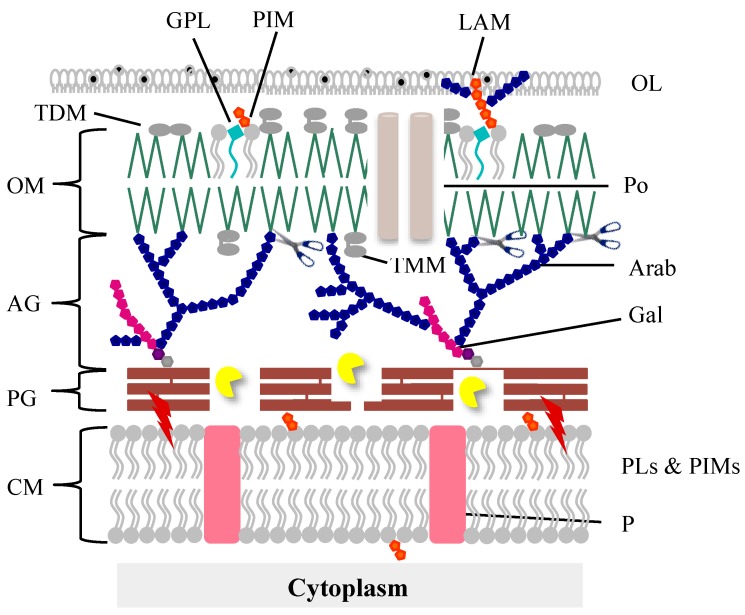
Schematic illustration of the mycobacterial cell envelope layers. The lysis proteins targets are indicated as follows: the red lightning bolts illustrate membrane depolarization by holins; the pacman cartoon represents digestion of the PG by the endolysins; scissors illustrate LysB detachment of the OM. AG, arabinogalatan; Arab, arabinan; CM, cytoplasmic membrane; Gal, galactan; GLP, glycolipids; LAM, lipoarabinomannan; OL, outer layer; OM, outer membrane; P, protein; PG, peptidoglycan; PIMs, phosphatidylinositol mannosides; PLs, phospholipids; Po, porin; TDM, trehalose dimycolate; TMM, trehalose monomycolate.

The mycobacteria cell envelope is a complex structure with specific characteristics distinct from both Gram-positive and Gram-negative bacteria that make it unique among the bacterial world. Its very high content in lipids (up to 60% of the CW is made of lipids) accounts for the highly hydrophobic cell surface properties, resulting in a natural impermeability to nutrients, resistance to many antibacterial drugs, and exceptional inflammatory activity, playing a major role in virulence [31]. The cell envelope (Figure 1) is composed of a CM, which is structurally and functionally similar to that of other bacteria [32]. This membrane is surrounded by the CW which is composed of a thin layer of PG covalently linked to an arabinogalactan (AG) polymer which is in turn esterified to mycolic acids (MA) forming the CW core known as the mycolyl-arabinogalactan-peptidoglycan complex (mAGP). A periplasmic space separates the CM from the CW. The MAs, one of the hallmarks of mycobacteria, are long chain fatty acids and are part of the inner leaflet of a membrane bilayer, a true OM [33,34], also known as mycomembrane. The outer leaflet of this membrane, structurally different from the OM of Gram-negative bacteria, is presumably composed of free lipids (noncovalently linked): phospholipids, glycopeptidolipids, trehalose mono (TMM), and di-mycolates (TDM)-an important virulent factor in pathogenic bacteria-and lipoglycans [31,35]. Finally, an outermost layer (OL), also called the capsule in the case of pathogenic species, is mainly composed of polysaccharides and proteins, with a minor amount of lipids, whereas in nonpathogens this OL is primarily constituted of proteins [36,37,38].

This complex structure is obviously a challenge to mycobacteriophage-mediated lysis and it seems evident that for their own survival, mycobacteriophages had to evolve specific lysis systems to be able to overcome each cell barrier and release the progeny virions at the end of a lytic cycle.

## 3. The Mycobacteriophage Lysis Proteins

The best-studied lysis system of a mycobacteriophage is that of phage Ms6, a temperate phage that infects the nonpathogen *Mycobacterium smegmatis* [24]. The lysis cassette of Ms6 contains five genes, designated *gp1* through *gp5* [39]. *gp1* codes for a chaperone-like protein that assists export of the endolysin (LysA) using the host *sec* system, in a holin-independent manner [40]. Gene *gp3* (*lysB*) encodes a specific lysis protein (LysB) with lipolytic activity, which is involved in disruption of the mycobacteria OM [41,42]. The proper time of lysis is ensured by the concerted action of two holin-like proteins encoded by genes *gp4* and *gp5*. Gp4, showing characteristics of a class II holin (small protein with two transmembrane domains), interacts with Gp5 (with characteristics of class III holins, i.e., with only one transmembrane domain) and it was proposed that this complex regulates the timing of lysis [43].

Not all mycobacteriophage lysis cassettes contain these five genes. *gp1* homologues are not present in most genomes and many contain only one holin-like gene. In some, a holin gene was not identified and a few lack *lysB* genes while LysA coding genes have been identified in all genomes [21,25]. Nevertheless, similarly to phages of Gram-negative hosts and due to the presence of an OM, it seems clear that mycobacteriophage-induced lysis is also a three-step event beginning with holins activity to subvert the CM, which leads to activation of the endolysin function to degrade the PG meshwork. The last step, i.e., the elimination of the OM, is played by LysB, which cleaves the linkage between the OM and AG (Figure 1).

Under the scope of this review, the mycobacteriophage endolysins and LysB proteins function will be described in more detail in the next sections.

## 4. Mycobacteriophages Endolysin (LysA)—The Essential Lysis Player

Endolysin is a wide denomination used to describe a variety of dsDNA bacteriophage-encoded PG hydrolases, which are synthesized in phage-infected host cells at the end of the replicative cycle [44]. They are unvaryingly characterized by their ability to directly target specific bonds in the PG layer of the bacterial CW; the result of this activity is degradation of the cross-linked PG meshwork in order to allow the release of the newly assembled phage particles progeny [10,44]. Phage endolysins should be clearly distinguished from other PG hydrolytic enzymes, the virion associated lysins (VAls) which are integral components of the virions’ tail that locally digest the CW from the outside to enable the phage genome to be injected into the host cell; an example of such an enzyme is the tail lysozyme of bacteriophage TM4 [45]. Within the phage genome endolysins are localized in a lysis casssete together with other lysis proteins, while VALs are usually associated with structural genes [46]. When inspecting the products encoded in a mycobacteriophage genomic sequence, possibly the only lysis protein whose function can be unequivocally assigned and is present in all genomes is the one corresponding to the endolysin [25,47]. In addition, the PG hydrolase activity of mycobacteriophage endolysins is the only lytic function essential and absolutely required for mycobacteria lysis; mycobacteriophages devoid of the endolysin activity are not viable as they are unable to complete the lytic cycle [48,49,50].

Mycobacteriophages have dedicated endolysins that have been predicted to target nearly every bond in mycobacterial PG (Figure S1 as previously published [25]). A bioinformatic analysis of 224 mycobacteriophage LysAs has identified six major types of catalytic activities: (i) *N*-acetyl-β-d-muramidases (lysozymes or lytic transglycosylases that form a cyclic 1,6-anhydro-*N*-acetylmuramic acid product) cleaving the β-(1,4) glycosidic bond between *N*-acetylmuramic acid and *N*-acetylglucosamine; (ii) *N*-acetyl-muramoyl-l-alanine amidases, which hydrolyze the amide bond between the *N*-acetylmuramic acid and l-alanine residues in the oligopeptide cross-linking chains; (iii) l-alanoyl-d-glutamate (LD) peptidase; (iv) γ-d-glutamil-meso-diaminopimelic acid (DL) peptidases; (v) d-ala-mDAP (DD) endopeptidase; and (vi) m-DAP-m-DAP (LD) endopeptidase. None of the 224 endolysins were predicted to contain an endo-β-*N*-acetylglucosaminidase activity [47] in accordance with the reported for other phage endolysins [13]. They are highly diverse and widely modular in their structure [47,51] and are generally composed by, at least, two well-defined regions: an N-terminal catalytic domain and a C-terminal PG-binding domain, with the majority containing an additional central catalytic domain [47] (see Section 4.2).

### 4.1. Structural Variations of the Mycobacterial Peptidoglycan

A hallmark of the mycobacterial CW is its unusual PG layer which belongs to the A1γ chemotype according to the classification system of Schleifer and Kandler [52]. Besides providing shape, rigidity, and counteracting osmotic pressure to mycobacteria bacilli, the PG contains a number of unique subtleties to the monomeric units, and other structural aspects that are possibly linked to a role in stabilizing the mycobacterial mAGP [53]. Mycobacterial PG is part of the mAGP complex and forms its basal layer. It is composed by the glycan chain which consists of alternating *N*-acetylglucosamine (GlcNAc) and modified muramic acid (MurNAc) residues linked in a β-(1,4) configuration; however, contrary to what is observed for other bacterial PG, the MurNAc residues in mycobacteria contain a mixture of *N*-glycolylated (MurNGlyc) or *N*-acetylated (MurNAc) derivatives where the muramic acid is preferentially hydroxylated to a *N*-glycolyl function by the action of NamH hydroxylase [54]. The *N*-glycolylation of MurNAc is uncommon, only present in mycobacteria and other closely related actinobacteria [55]; although the precise role of the *N*-glycolyl function is yet to be elucidated, it has been hypothesized that it contributes to the lysozyme-resistant phenotype of mycobacteria, promotes the recognition of PG by the host innate immunity protein Nod2, and may potentiate additional hydrogen bonding interactions, strengthening the meshwork structure of the PG layer [54,56]. The tetrapeptide side chain attached to the lactyl moiety of MurNAc typically consists of l-alaninyl-d-isoglutaminyl-*meso*-diaminopimelyl-d-alanine, cross-linked to identical short peptides of neighboring glycan chains [53]. Variations in the tetrapeptide include amidation of the free carboxyl group of d-Glu (generating d-*iso*-Glu), and of the free carboxyl of the side chain of *meso*-diaminopimelate [57]. Cross-linking is catalyzed by d,d-transpeptidases (penicillin-binding proteins) and mostly by the concerted activity of nonclassical l,d-transpeptidases (Ldts) and d,d-carboxypeptidases resulting in 4→3 (d-Ala-mDAP) and 3→3 (mDAP-mDAP) linkages, respectively [53,58]. Mycobacteriophage PG hydrolases are associated with extensive genetic multiplicity suggesting functional diversification within this family of enzymes to allow mycobacteriophages to adapt to the peculiar mycobacterial PG. Considering this, it seems that the highly modular nature of mycobacteriophage endolysins reflects the atypical cross-linking types and modifications of mycobacterial PG.

### 4.2. LysAs Structural Diversity and Modular Organization

**Figure 2:**
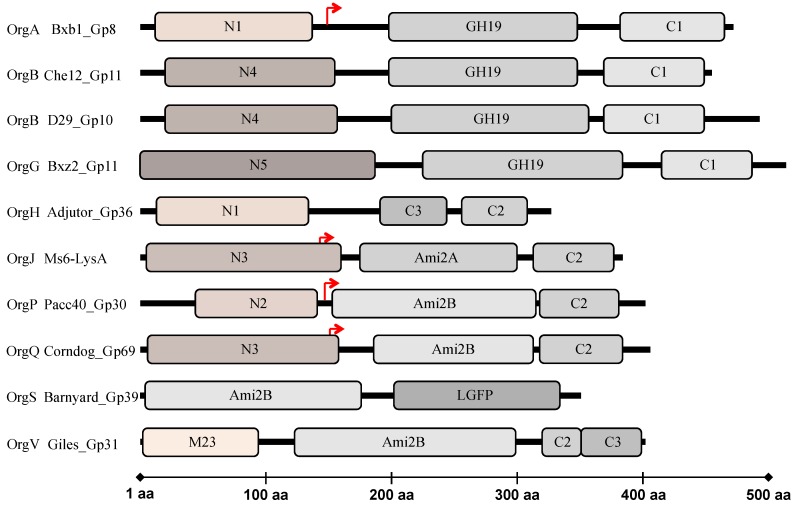
Modular organization of mycobacteriophage’s LysA proteins. Illustrated are representatives of mycobacteriophage endolysins with different domain combinations. The three predicted domains are differently colored. Putative peptidases: N1–N5 and M23 are shaded in beige; catalytic domains are marked with strip patterns; CWBDs are shaded in grey and LGFP repeats (suggested to be involved in cell wall anchoring) in dark grey. The red arrows indicate the start of the truncated versions of LysA. Coordinates of each domain were obtained from table S1 in a previous paper [47]. Protein accession numbers are: Bxb1_Gp8(AAG59713), Che12_Gp11(ABE67330), D29_Gp10(AAC18450), Bxz2_Gp11(AAN01764), Adjutor_Gp36(ACD49621), Ms6_LysA(AAG48318), Pacc40_Gp30(ACI12541), Corndog_Gp69(AAN02001), Barnyard_Gp39(AAN02093), and Giles_Gp31(ABW88426). Adapted from a previous work [47].

Notwithstanding the vast diversity of the mycobacteriophage endolysins, encompassing remarkable size variation and intragenic mosaicism, there are common features in regard to their organizational structures. As mentioned above mycobacteriophage endolysins were shown to have a modular organization [47], similarly to the endolysins from phages infecting Gram-positive hosts and in contrast with most endolysins that target Gram-negative bacteria, which have usually a globular nature [12,59,60]. Most of them (∼90%) are composed of three conserved segments [47] in contrast to most of other phage endolysins with a conserved modular structure which are composed of two clearly separated functional domains: the N-terminal domain which generally harbors the enzymatic activity, whereas specific cell wall binding domains (CWBD) located at the C-terminal direct the enzymes to their substrates restrain the enzyme lytic action to a particular type of cell [12,60]. The three-segments of mycobacteriophage endolysins, include a C-terminal domain that functionally resembles the C-terminal CWBD of other phage endolysins, a central domain that usually encompasses a conserved motif with amidase, muramidase, or transglycosylases activity involved in PG degradation, and an N-terminal domain that was proposed to encode a variety of peptidases targeting the PG bonds within the tetrapeptide chain and the atypical inter-peptide cross-links [47] (Figure 2). However, there is significant diversity among members of each domain and numerous endolysins structures depart from this general organization; the bioinformatic analysis identified six possible types of N-terminal peptidase domains, five types of amidase/glycosidase domains, and four putative C-terminally located CW binding motifs [23,47]. In total, there are at least 25 different endolysin organizations (Org-A to Org-Y) with distinctive combinations of the composing domains. Some of these follow a different domain organization presenting two CWBD (Org-D, H, T, and V) or none (Org-L), and others do not present the glycosidase or amidase catalytic domain (Org-C, H, M, T, and Y) [47]. The structural diversity and modular organization of mycobacteriophage endolysins is atypical and reflects the adaption of these viruses to the complex CW of their mycobacterial hosts.

Despite being classified as Gram-positive bacteria, mycobacteria possess an OM as in Gram-negative bacteria. It has been suggested that differences in modular organizations between endolysins from Gram-positive and Gram-negative could be related to the absence or presence of this structure. It has been proposed that the separated CWBD in endolysins from Gram-positive bacteria have an important role in keeping the enzyme tightly bound to the cell debris after cell lysis [12]. This would prevent diffusion of the enzyme in the environment, which, if free, could act exogenously on eventual neighboring available cells for new infective cycles. In Gram-negative hosts the PG is protected by an OM, limiting the access of free endolysins, explaining the unnecessary presence of a specific cell wall binding domain. In mycobacteria the PG layer is also protected by an OM, thus there is no clear explanation for the necessity of separate CWBD. It should however, be pointed out that the cell binding capacity of these domains was not, as yet, experimentally demonstrated.

### 4.3. Mycobacteriophage LysAs Catalytic and Binding Domains

As referred previously, mycobacteriophage PG hydrolases have evolved to adapt to the specific characteristics of the mycobacterial PG. The enzymatic activities that cleave the β-(1,4) linkages between the MurNAc and GlcNAc moieties of the PG glycan strand are usual among bacteriophage-encoded endolysins and are well-represented amid the mycobacteriophage endolysins [47]. These are characteristically present as N-terminal or central domains of the enzymes and encompass *N*-acetyl-β-d-muramidases belonging to the glycoside hydrolase family GH25 (cd06523) or GH19 (cd00325) which were predicted to be present in several mycobacteriophage endolysins [47]. GH25 activity is a common muramidase (lysozyme-like) among phage endolysins that cleaves β-(1,4) linkages between the MurNAc and GlcNAc residues. GH19, classified as a chitinase domain, has been identified in LysAs from diverse mycobacteriophage clusters mainly in members of the cluster A such as Che12. Che12 LysA was predicted to act on NAG-NAM-NAG trimer in the PG glycan chain and encompasses a motif of eight amino acids, G-R-[DGT]-X-I-Q-[IL]-[ST], conserved in 19 endolysins from mycobacteriophages belonging to clusters A, B (Rosebush and Qyrzula), K (CrimD and Angelica), and V (Wildcat) [61]. Proteins having chitinase-like domains generally act on β-glycosidic linkages, which are present in chitin and in PG; interestingly, although nearly all mycobacteriophages have a LysB protein, there are a few examples in which the LysB is absent. In the study performed by Payne and Hatfull [47], all phages where a *lysB* gene was not predicted, with the exception of Myrna, encoded a LysA protein with a GH19 domain. However, at this time there is no evidence that a chitinase-like endolysin compensates for the lack of LysB.

The transglycosylase (TG) activity (pfam06737) included in the lysozyme-like superfamily seems to be under-represented among mycobacteriophage endolysins, being restricted to phages within Cluster A, where the TG is the unique central catalytic domain, flanked by conserved N-terminal and C-terminal domains [47]. Given the uncommonness of TG activity found thus far in mycobacteriophage, TG-containing endolysins are interesting variations of the usual catalytic activities; preliminary data on the Ms6 endolysin suggests that this TG activity may be specific for *N*-glycolylated PG [62].

As reported by Payne and Hatfull [47], the mycobacteriophage endolysins containing domains related to the amidase-2 conserved domain (pfam01510) are over-represented among 224 phage endolysins, presenting a wide range of sequence diversity. Phylogenetic analysis showed that the Ami-2 domain fall into two major clades, referred to as amidase-2A (Ami-2A) and amidase-2B (Ami-2B) domains. The Ami-2A domain is slightly more predominant than the Ami-2B domain and both fit within the larger superfamily of PGRP, PG-recognition proteins (cd06583) that recognize and hydrolyze the PG [47]. In the same study, several peptidase domains were predicted within mycobacteriophage endolysins, some of which are novel and have not been reported in other phage endolysins. A total of six sequence variants have been proposed: the M23 peptidase domain and N-terminal domains N1–N5. The N1 domain appears to encode an l-Ala–d-Glu peptidase activity, which has been found in endolysins of phages infecting hosts that possess type A1γ PG, suggesting substrate specificity. The N2 and N3 domains are likely to be distant relatives of M23 peptidases presenting high similarity to the Gp13 tail knob protein of phi29, which has a metalloprotease domain of the M23 family. The N4 domain presented similarity to CW-associated mycobacterial proteins including *M. tuberculosis* RipA (Rv1477), a peptidoglycan hydrolase that interacts with resuscitation-promoting factors; RipA has a peptidase active site that cleaves between d-Glu and m-DAP residues. The N5 domain matches several cysteine proteases of *Staphylococcus aureus* of the papain family [47]. There are a number of potential peptidase targets within the mycobacterial PG, both within the stem tetrapeptide chain and at the interpeptide bridges; endolysin peptidase activities are likely to be particularly informative about these structures. It should be noted that these enzymatic activities were bioinformatically predicted, and demand further confirmation as the N-terminal domain of the mycobacteriophage Ms6 endolysin was experimentally shown to lack the predicted peptidase-like activity [62].

In addition to the catalytic domains, CWBD were also suggested to be part of mycobacteriophage endolysins. Four different types of C-terminal sequence motifs were identified, being C1, C2, and C3, the three more common. Only the endolysins with a C3 motif are predicted to have a peptidoglycan binding domain (pfam01471). Although no conserved domains were identified in C1 and C2 motifs, Payne and Hatfull [47] admit a role in cell wall recognition. The fourth cell wall associated domain is a repeat of a LGFP motif (pfam08310), observed in the endolysin of mycobacteriophage Barnyard. All analyzed endolysins in this study [47] are synthesized by mycobacteriophages that infect the same host (*M. smegmatis*), as the majority of all isolated mycobacteriophages, and their host range is not known. However it is expected that these phages infect other mycobacteria species and these CWBD are likely to be involved in specific recognition of the mycobacterial CW.

The complexity of the mycobacteriophage endolysins is remarkable, encompassing interchangeable domains assembled into an enormous diversity of structural organizations; presumably there is a strong selective advantage to the exchange of these modules, in response to specific modifications in host PG that confer resistance to the action of these endolysins, or the ability to allow the effectiveness of lysis in an alternative host.

### 4.4. Mycobacteriophage LysAs Expressing Secondary Gene Products

The use of intragenic secondary translation sites as a regulation switch is widespread among bacteriophages lysis modules and there are several cases of fully or partially overlapped genes in phage genomes. Alternative start sites have been described for several endolysin genes: examples include the endolysins encoded by the *S. aureus* phage 238A [63], *Enterococcus faecalis* phage F170/08 [64], and mycobacteriophages Ms6 [50] and Corndog [47]. This endolysins noncanonical feature may reflect the need for additional hydrolytic activities and/or binding domains to efficiently target the several bonds within the PG meshwork and phages have adapted in order to encode and regulate endolysins responsive to more complex PG substrates with higher degree of cross-linking or unusual interpeptide linkages.

The first report of in-frame secondary translation within a mycobacteriophage endolysin came from the work of Catalão et al. (2011) [50] on the Ms6 endolysin. Ms6 LysA displays a conserved modular architecture of two separated functional regions: a central catalytic motif holding a PG-recognition protein (PGRP) conserved domain, localized between amino acid residues 168 and 312 [50] with *N*-acetylmuramoyl-l-alanine amidase activity that cleaves the bond between the d-Mur and l-ala of the stem peptide in the highly amidated mature PG [65], linked to a C-terminal region presumably involved in CW binding [47,50,51]. The N-terminal region of Ms6 LysA, predicted to contain a N3 domain [47] binds the Gp1 secretion chaperone. Gp1 assists the enzyme access to the PG by somehow allowing its translocation across the cytoplasmic membrane independently of Ms6 holin-like protein activity [40]. Remarkably, the Ms6 *lysA* gene encodes two products, designated Lysin_384_ and Lysin_241_ according to the size of the polypeptides synthesized: Lysin_241_ is produced from a second translation initiation codon in the same reading frame of Lysin_384_ (*lysA*), positioned 143 amino acids far from the first initiation codon (Figure 2). Ms6 devoid of *lysA* is not viable, suggesting that LysA PG hydrolase activity is absolutely required to induce host cell lysis [50]. Similar results were observed for other mycobacteriophages: disruption of phage Giles LysA (Gp31), results in loss of phage release without affecting virions assembly [49]. An intriguing observation was that only one of the Ms6 LysA peptides was shown to be required for mycobacterial lysis. Notwithstanding, the normal timing, progression, and completion of host cell lysis was visibly affected; lack of Lysin_384_ resulted in a lysis delay of 30 min and in a reduction in the number of phage particles released, while in the absence of Lysin_241_ lysis starts 90 min later with no significant effect in the number of phage particles released [50], which strongly indicates that both enzymes are necessary for complete and efficient degradation of the mycobacterial PG. These results were supported by studies on the mycobacteriophage Corndog endolysin (Gp69) that has a similar gene arrangement, with a second translation initiation codon at position 147 resulting in the production of two distinct proteins, a truncated and a full-length enzyme (Figure 2), where the former seems to be more active [47] (see Section 4.6). The prevalence of internal gene initiation codons that result in the synthesis of two endolysins in the lysis modules of some mycobacteriophages may therefore represent an evolutionary advantage over mycobacteriophages producing only one PG hydrolase.

### 4.5. LysAs Delivery to the Mycobacterial Peptidoglycan

Mycobacteriophages use the so-called holin-endolysin strategy to achieve lysis of the mycobacterial hosts as the vast majority of bacteriophages described to date [10]. Endolysins are produced in the cytoplasm during the latent period and to gain access to the PG layer they have to cross the CM. Canonical endolysins achieve their target through the holes formed by canonical holins. Noncanonical endolysins are translocated in a holin-independent manner as they are synthesized, remaining inactive in the extracytoplasmatic environment, to prevent premature lysis, until holins triggers depolarization of the CM. These endolysins contain N-terminal secretion signals (signal peptides (SP) or signal-anchor release (SAR) sequences) and use the host *sec* system for membrane translocation [66,67,68,69,70]. All of the mycobacteriophage endolysins identified so far lack SP or SAR sequences to be exported suggesting an entirely dependence on the holin activity for cell wall access at the end of a phage the lytic cycle [10,25]. Notwithstanding, an alternative holin-independent delivery of a LysA protein has been reported for mycobacteriophage Ms6 [40].

Despite not being endowed with a predicted secretion signal, the export of the Ms6 endolysin is assisted by a chaperone-like protein, produced from gene *gp1* [40]. As described for other secretion chaperones, Ms6 Gp1 interacts with its effector, the LysA protein, and binding requires the N-terminal region of the chaperone and the first 60 amino acids of the Ms6 endolysin [40,70]. Even though the Ms6 *lysA* gene encodes two proteins, Lysin_384_ and Lysin_241_, only Lysin_384_ interacts with Gp1; although the N-terminal region that interacts with Gp1 is absent in Lysin_241_, this truncated version keeps the enzyme catalytic domain [50]. It was suggested that secretion and stabilization of Lysin_384_ relies on the specific binding of Gp1 to Lysin_384_ as in the absence of the chaperone, the enzyme stability is severely affected during infection of *M. smegmatis*. A deletion of *gp1* from the Ms6 genome revealed that, although not essential for plaque formation, Gp1 is necessary to achieve an efficient lysis, since its absence resulted in a marked decrease in the burst size [40] possibly due to inefficient cleavage of the mycobacterial PG as a result of Lysin_384_ degradation. *gp1* homologues have been identified in other mycobacteriophage genomes, particularly in the lysis cassette of phages that belong to subclusters A1 and F1 [71] and in phage Charlie, a member of cluster N [10,71]. One exception is *gp90* of phage TM4, from subcluster K2, which is positioned outside the lysis cassette [72]. *gp1* homologues seem to be missing in several mycobacteriophage genomes, suggesting that in these phages endolysin export departs from the Ms6 lysis strategy.

Although secretion of the endolysin to the extracytoplasmatic environment is not dependent on the holin function, this is still necessary to determine the timing of lysis. Mycobacteriophage Ms6 regulation of mycobacteria lysis timing seems also to display peculiar and complex features. Host cell lysis at the correct and programmed timing was shown to be dependent on the interaction and concerted action of two membrane proteins with holin-like characteristics even though they individually are not required for phage viability [43]. The gene product encoded by *gp4* was identified as the Ms6 holin as it shares structural features with class II holins and is able to complement an S^λ^ defective mutant [39,43]. A more detailed characterization of Ms6 Gp4 activity suggested that Gp4 might function as a pinholin, as the first TMD has characteristics of a SAR domain with a high percentage of weakly hydrophobic or polar uncharged residues [43,73], followed by a typical TMD. In Ms6, a second putative holin-like protein, *gp5*, was found to be positioned immediately downstream of the *gp4* gene [39,43]. Although an infection of *M. smegmatis* without Gp5 results in a delay of the timing of lysis, Gp5 did not complement an S^λ^ defective mutant. It was proposed that *gp4* and *gp5* encode holin proteins whose combined action might play the role of a holin, as expression of both proteins is necessary to achieve host cell lysis at the correct and programmed timing. Moreover, interaction of Gp5 with Gp4 contributes to the precise adjustment of the timing of hole formation allowing a productive infection cycle [10,43].

At this time it is unknown if Lysin_384_ activation is dependent on the holin function and how Lysin_241_ accesses the PG; a role for Gp1 in maintaining Lysin_384_ in an inactive state until membrane depolarization by the concerted action of the Ms6 holins has been proposed but demands further clarification.

Although a holin-independent export has only been demonstrated for the Ms6 phage, it is expectable that Ms6 relatives (subcluster F1 members), having homologous lysis cassettes [10,71], use the same strategy to lyse mycobacterial hosts. As mentioned above, mycobacteriophages may have simpler lysis cassettes with one *lysA* gene, one holin, and one *lysB* gene. The diversity on the lysis cassette composition suggests different models for host lysis, particularly in what concerns the endolysin export and activation.

Holin-independent lysis mediated by some LysA proteins has also been reported [26,40,47]. Expression of D29_Gp10, L5_Gp10, and Kostya_Gp33 from a mycobacteria plasmid, caused a high intracellular ATP-release three hours after induction due to cell lysis. Although lysis occurs efficiently in the absence of the holin gene it is unlikely that during phage infection lysis occurs entirely in a holin-independent way as all the three phages encode in their genomes holin-like proteins with predicted membrane-spanning domains [47]. A reasonable explanation is that high levels of LysA expression and interaction with the cytoplasmic membrane cause sufficient destabilization of the membrane potential that enables endolysin access to the PG substrate [26,47].

For a better comprehension of mycobacteriophage endolysins activity, investigation on mycobacteriophage-induced lysis should be highly encouraged.

### 4.6. Regulation of Mycobacteriophages Endolysin Activity

Several molecular mechanisms of endolysin activation have been reported. For the SAR endolysins, which remain inactive in the periplasm by anchoring the SAR domain to the CM, activation relies on protein refolding after membrane releasing following the holin trigger [67,68,74]. Mycobacteriophages, and phages that infect Gram-positive bacteria, are known to produce endolysins with multiple domains [14,44]. These enzymes usually contain one or two catalytic domains at the N-terminal end and a CW-binding domain toward the C-terminal region. In many phages, endolysin activity is regulated by secondary translation products. The *lys170* gene of the enterococcal phage F170/08 [64] and the CTP1L coding gene of the *Clostridium* phage φCTP1 [75] have been shown to encode two polypeptides, the full-length endolysin and a C-terminal fragment corresponding to the CW-binding domain, the latter resulting from an in-frame, secondary translation start site. Assembly of the functional endolysin requires the interaction of both peptides, which are necessary for efficient binding of the endolysin to its substrate [64,75]. The appropriate ratio between the full-length protein and the secondary product seems to be determinant for lysis efficiency. It was speculated that the oligomeric switch resulting from secondary translation events, may constitute a novel strategy used by phages to increase the number of CW motifs [75] as an alternative to the CW binding tandem repeats commonly found in pneumococcal phage endolysins. It has been proposed that the number of CWBD have an important role in the binding of the enzyme to the cells, regulating the catalytic activity [64,75]. The endolysin from the staphylococcal phage 2638A has a similar domain architecture to most mycobacteriophage endolysins, with a N-terminal peptidase domain, a central amidase domain, and a C-terminal binding domain. In this case the secondary translation occurs between the two catalytic domains generating a second peptide that includes the amidase and CWBD, similarly to what is observed for the Ms6 Lysin_241_. The authors have reported that a mutation abolishing the secondary product results in a decreased lytic activity [63].

As mentioned, Ms6 and Corndog phages also translate two polypeptides from the *lysA* gene as a result of in-frame internal initiation start codons [47,50]. Similarly to CTP1L the additional synthesized polypeptides are truncated versions of LysA lacking the putative peptidase N-terminal domain and conserving the Ami_2 central domain and the predicted CW binding domain (C2) (the Lysin_241_ in Ms6) (Figure 2), and similar differences in the enzyme lytic activity have also been reported. It has been suggested for the Corndog Gp69 endolysin that the N3 peptidase N-terminal domain may have a regulatory role, inhibiting the amidase catalytic activity of the full-length protein, since it was observed that the truncated Ami2B-C2 form of Corndog Gp69 presents higher activity in a zymogram assay [47]. It is not known however, if both proteins are required for a productive phage infection cycle. In the Ms6 endolysin case, it has been postulated that the truncated Lysin_241_ has an important function in the progression and completion of host cell lysis and may compensate for Lysin_384_ instability notwithstanding the fact that Lysin_384_ is necessary to achieve a normal burst of infective phages [50]. In addition, Gp1 binding to Lysin_384_ during secretion may have a role in keeping Lysin_384_ in the proper conformation and/or protection from degradation [40]. Many other mycobacteriophage *lysA* genes present a potential second translational site suggesting that similarly to bacteriophages that infect Gram-positive bacteria, the use of secondary translated products regulating the endolysin activity may be a common phenomenon among mycobacteriophage endolysins.

The regulation of the mycobacteriophage D29 LysA activity has also been dissected by Pohane et al. [26]. The authors showed that the enzyme harbors two catalytic domains for PG hydrolysis, an N-terminal peptidase domain and a central glycoside hydrolase (lysozyme) domain. Interestingly, expression in *M*. *smegmatis* of the N-terminal putative peptidase domain and the full-length protein resulted in cell lysis upon protein expression induction; however, no similar effect was observed when only the lysozyme domain was expressed in mycobacteria. Surface plasma resonance experiments showed that the N-terminal and C-terminal domains of the D29 endolysin interact, and that this interdomain interaction renders the enzyme inactive in *E. coli* (see Figure 10 of a previous paper [26]). Moreover, this interaction is abolished in *M. smegmatis* most likely due to PG that competes for D29 endolysin C-terminal binding domain which results in the activation of LysA; such interaction was proposed to make the endolysin bacteria-specific [26]. In another study, the same authors proposed that the D29 LysA interdomain interaction occurs intramolecularly and is facilitated by a charged linker that connects the two domains [27]. In addition, the central catalytic domain that has been predicted to possess glycoside hydrolase (GH19) activity was shown to possess a residue R198, positioned away from the catalytic site and not directly involved in substrate binding, which plays a critical role in the maintenance of the substrate-binding cavity [76]. Taken together these data indicate that mycobacteriophage endolysin activity regulation may be more complex than previously anticipated, involving intramolecular domains interaction and synthesis of secondary gene products in order to optimize mycobacteria CW binding and enzyme activation; these mechanisms may improve PG breakdown which will culminate in optimal bacterial cell rupture and phage release.

## 5. Mycobacteriophages LysB: Essential for Effective Disruption of the Last Barrier to Phage Release

In 325 out of 350 complete genomes available in GenBank, a *lysB* gene was clearly identified. In 25 genomes, a *lysB* gene was not bioinformatically predicted or annotated as so. When present, it was always localized within the lysis module, downstream of the *lysA* gene. This suggested that LysB would play a role in lysis. A multiple sequence alignment of LysB proteins have shown that they are highly diverse with some proteins showing less than 20% of identity to each other [49]. This diversity is not restricted to phage cluster as the same Pham may group LysB proteins from different phage clusters (Table S1). However, they all have in common a G-X-S-X-G motif that is characteristic of lipolytic enzymes [41,49,77]. A lipid hydrolase function of a LysB protein was first demonstrated for the one encoded by the mycobacteriophage Ms6. A biochemical characterization has shown that Ms6 LysB is capable of hydrolyzing both esterase and lipase substrates within a wide carbon length range [41]. Evaluation of the kinetic parameters for the hydrolysis of *p*-nitrophenyl esters showed a higher affinity for the longer chain length substrates (C16 and C18), being consistent with the presence of long carbon chain lipids in the cell envelope of mycobacteria. The highest specific activity (0.12 U/mg) observed with *p*-nitrophenyl butyrate (pNPB) (C4) was enhanced almost twice in the presence of Ca^2+^, suggesting that for future applications addition of this divalent cation will improve activity [41]. Ca^2+^ has been shown to increase the activity of lipolytic proteins, stabilizing and activating these enzymes [78].

A lipolytic activity was also reported for the D29 LysB, a protein that shares only 40% identity with Ms6 LysB. The specific activity on pNPB was 0.72 U/mg, somewhat higher than the observed for Ms6 LysB [49]. The crystal structure of D29 LysB was already determined and revealed to be similar to cutinases, folding as the α/β hydrolase superfamily of proteins. Cutinases, lipases, and esterases are all lipolytic enzymes, members of the serine hydrolase family that fall into the large α/β fold superfamily (for a more detailed information see previous studies [78,79,80]). The activity of these enzymes relies on a catalytic triad formed by Ser, Asp (or Glu), and His residues in this order in the amino acid sequence, with Ser being part of the pentapetide G-X-S-X-G [78,79]. The 3D structure of D29 LysB identified Ser82, Asp166, and His240 as the residues that form the catalytic triad. A multiple alignment of several LysB proteins including D29 LysB shows that the Ser residue is absolutely conserved. The Asp or Glu are also highly conserved, while for His, some variation is observed. Many His residues are very well conserved although displaced 1 or 2 residues from D29 LysB His240, showing to be good candidates to be part of the catalytic triad (Figure S2).

The natural target for Ms6 and D29 LysB was already reported. Both proteins were shown to cleave the ester bond that links the MA present in the inner leaflet of the mycobacteria OM and the CW PG-AG polymer, separating the OM from the CW [42,49]. In addition to being part of the OM, MAs are also part of other lipids of the mycobacteria cell envelope such as the trehalose dimycolate (TDM). TDM is a glycolipid that is present in all mycobacteria playing an important role in virulence of pathogenic mycobacteria. Importantly, the Ms6 LysB was shown to hydrolyze the TDM of the nonpathogenic *M. smegmatis* as well of *M. bovis* BCG and *M. tuberculosis* H37Ra [42] showing a potential to reduce virulence in *M. tuberculosis*. It has already been shown that although mycobacteria can survive without TDM, its absence leads to a reduced viability of mycobacteria inside macrophages [81]. Taking together the reported analysis of Ms6 and D29 LysB, it seems evident that these enzymes, although sharing little amino acid sequence identity, share the same catalytic activity.

In addition to the identification of the amino acid candidates to form the catalytic triad, alignment of D29 LysB with other LysB shows that many proteins have an extended N-terminus, which is not present in D29 LysB (Table S1). That is the case of Ms6 LysB and other LysB proteins (Figure S2). Previous analysis of the amino acid sequence of LysBs has predicted a PG-Binding Domain (PGBD) (pfam01471) within this region in some members of the LysB family of proteins. Although not predicted for Ms6 LysB, the region encompassing amino acids 1–90 has PG binding ability [82]. It is not clear, at this time, what is the advantage of this PGBD in LysB to mycobacteria lysis.

The importance of LysB proteins in lysis was shown by Payne et al. (2009) [49] and Gigante et al. (2017) [83]. Similarly to spanins, which are now known to be essential for lysis of Gram-negative hosts, mycobacteriophage LysB proteins seem also to have a relevant role in host lysis. Although not shown to be essential for lysis in laboratory conditions, deletion of gene *lysB* from mycobacteriophages Giles or Ms6 results in a lysis defect, yielding a reduced number of phage particles. In fact, in a *M. smegmatis* infection with the mutant Ms6*ΔlysB* the burst size drooped 64% [83]. In an assay measuring the release of ATP, infection with Giles*ΔlysB* showed a delay of 30 min compared to Giles*wt* [49], while no differences in time of lysis was observed with Ms6*ΔlysB* [71].

Overall, the high diversity among the LysB proteins may account for differences in the catalytic activity and effectiveness in cell lysis.

Cryo-electron microscopy of mycobacteria cells infected with Ms6*ΔlysB* clearly showed that in absence of LysB the cells become deformed with phage particles trapped in incompletely lysed cells, while at the same time point cells infected with Ms6*wt* are completely lysed [83]. This supports the notion that the OM is an important barrier that still holds part of the cell content even after disruption of the CM and PG, and thus for an efficient lysis it must be disrupted. LysB proteins acting as hydrolytic enzymes have the potential to eliminate this barrier, having an important role in the final step of mycobacteriophage-induced lysis (Figure 1).

Of note is the fact that LysB homologues are also encoded in the genomes of phages infecting members of the mycolata group [81,84], i.e., bacteria that have a layer of MA attached to PG. This strengthens the notion that elimination of this layer is important for a productive phage infection.

## 6. Potential Value of Mycobacteriophage Lysis Enzymes

The potential of endolysins as antimicrobials has shown to be promising on Gram-positive hosts. For Gram-negative bacteria the OM imposes a natural barrier to the access of the enzymes to the PG, however several engineered endolysins are in development to circumvent this barrier. Access of the mycobacteriophage endolysins to the PG substrate is challenging due to the presence of the MAs-rich mycobacterial OM [33,34]. Nevertheless, mycobacteriophage Ms6 endolysins, Lysin_384_ and Lysin_241_, were shown to inhibit the growth of some mycobacterial species (*M. smegmatis*, *M. aurum* and *M. fortuitum*) when exogenously added despite their MA OM; it was postulated that LysA access to the PG may occur during cell division and septal PG synthesis [50]. In another study, the effect of mycobacteriophage BTCU-1 endolysin on *M. smegmatis* morphology and its ability to kill intracellular *M. smegmatis* was addressed. Scanning electron microscopy (SEM) showed that LysA-treated *M. smegmatis* displayed a wide range of significant abnormalities, including cell structure collapse with irregular spheroidal morphology. In addition, treatment of *M. smegmatis*-infected macrophages with BTCU-1 LysA resulted in a significant decrease in the number of viable intracellular bacilli [85]. The utility of mycobacteriophage-derived endolysins for mycobacterial PG structure analysis has also been study; this is of particular importance since the PG isolated from *Mycobacterium* spp. is refractory to the most commonly used commercially available murolytic enzymes, possibly due to the presence of secondary modifications of the mycobacterial PG, namely the *N*-glycolylation of the muramic acid of the glycan strand, the amidation of the m-DAP carboxylic acid function, and the addition of a glycine or serine to the stem peptide [65]. Utilization of specific endolysins that target the complex mycobacterial PG may shed some light on the nature of these atypical modifications, their contribution for mycobacterial persistence, and the intrinsic resistance to some class of antibiotics that target PG synthesis, such as the beta-lactams which thwarts their use as an alternative in TB treatment.

Notwithstanding the observation that the use of mycobacteriophage endolysins for therapeutics are not yet a possibility, some of these enzymes may be of practical utility in a variety of sceneries. Attempts to develop culture-independent diagnostic probes for mycobacteria and detection were successfully described recently; the Gp10 endolysin of mycobacteriophage L5 was shown to recognize and bind to the *M. smegmatis* cell surface and can be potentially used to improve the effectiveness of existing mycobacterial infections diagnostic platforms [86].

In what regards LysB proteins, although few studies have been performed, they have shown to have promising applications. In contrast to spanins that exert their function by a topological mechanism excluding the possibility of being used exogenously, LysB proteins act enzymatically, detaching the OM by cleaving their linkage to the PG-AG polymer. The studies performed with the LysB protein of mycobacteriophage BTCU-1 showed the same morphological modifications described above for the BTCU-1 LysA. When applied exogenously, BTCU-1 LysB was also active against *M. smegmatis* as observed for the LysA protein [85]. However how these enzymes access their targets to kill the cells is not clear and remains to be explained.

Preliminary data have shown that a previous treatment of mycobacteria cells with purified Ms6 LysB facilitates the binding of proteins, harboring a PGBD domain, to the cell surface [82], suggesting that the OM was sufficiently disturbed to allow decoration of the cells with PGBD containing proteins.

Therefore the use of LysB alone or in combination with mycobacteriophage endolysins or conventional antibiotics that target MA synthesis seems to be the most promising approach, particularly in the case of antibiotic-resistant bacteria. As mentioned above the Ms6 LysB was also shown to hydrolyze other MA-containing lipids, such as the TDM. These free lipids are part of the outer leaflet of the OM and as such are accessible to an external access and their hydrolysis would therefore, disturb the OM. In addition TDM is an important virulence factor for mycobacteria and LysB proteins are good candidates to explore their potential to reduce pathogenicity.

## 7. Conclusions

A hallmark of mycobacteria is their intricate cell envelope, essential for cell viability. Therefore, inhibition of its assembly has proven useful for tuberculosis chemotherapy and the same may be extrapolated for the enzymatic degradation of its components. In this way the lysis enzymes produced by mycobacteriophages, which target essential cell envelope structures seem to be promising candidates do destroy pathogenic mycobacteria.

Access of the mycobacteriophage endolysins to their substrate, the PG is thwarted by the MA-rich mycobacterial OM, which limits its use as therapeutic agents to fight tuberculosis. Therefore, entry of phage enzymes into mycobacterial cells remains the major confounding factor in the application of these proteins in therapy. Although growth inhibition was reported, the ability to kill the cells when added exogenously is not clear. As LysB proteins hydrolyze lipids on the external layer of the OM, in addition to a mycolyl-arabino-galactan esterase activity, one might envision the possibility of a synergy effect in combination with endolysins or even other antibiotics that have a limited access to their target and this should be explored.

The current developments on the knowledge of the mechanisms of mycobacteriophage-mediated lysis gave new insights in the way mycobacteriophages eliminate each layer of the mycobacteria envelope and are of relevance for the design of new strategies to compromise the survival of mycobacteria. In addition, given the plethora of mycobacteriophage endolysins enzymatic activities, adapted to degrade specific bonds within the mycobacterial PG, the possibility of using these in combination, such that a highly effective variety of enzymatic activities could be provided, is tempting and meritorious of further investigation. This is an advantage over the use of whole mycobacteriophage (Phage Therapy) because to date very few phages were shown to infect the tubercle bacilli. The vast number of isolated mycobacteriophages therefore represents an enormous reservoir of diverse CW degrading enzymes that are potentially useful for many applications in therapy and biotechnology.

An additional challenge is posed in what regards the application of such enzymes to an intracellular pathogen, as it is *M. tuberculosis*. Activity of an endolysin against intracellular bacteria was recently reported [87]. The authors have shown that PlyC, an enzyme from a streptococcal phage, can cross epithelial cell membranes and clear intracellular *Streptococcus pyogenes*. The more studies on endolysins exist, the more promising they look for the killing of pathogenic bacteria. Delivery to the local of infection will be the next hurdle to take. Several research groups are currently exploring the use of nanoparticles for delivery of antibiotics and other antimicrobial agents, including endolysins [88], which shows to be a promising system to get antimicrobials inside infected macrophages.

## Supplementary Materials

The following are available online at http://www.mdpi.com/1999-4915/10/8/428/s1, Table S1: Coordinates of LysB; Figure S1: Schematic representation of the endolysins targets in the bacterial peptidoglycan, Figure S2: Alignment of LysB proteins.
