# Supplementary material for: Mycobacteriophage Lysis Enzymes: Targeting the Mycobacterial Cell Envelope
Source: Viruses. 2018 Aug 14;10(8):428. doi: 10.3390/v10080428 (PMC6116114; doi:10.3390/v10080428)
Supplement: Supplementary file 1 [file viruses-10-00428-s001.zip › viruses-331689-supplementary-final check/Figure S1.pdf]

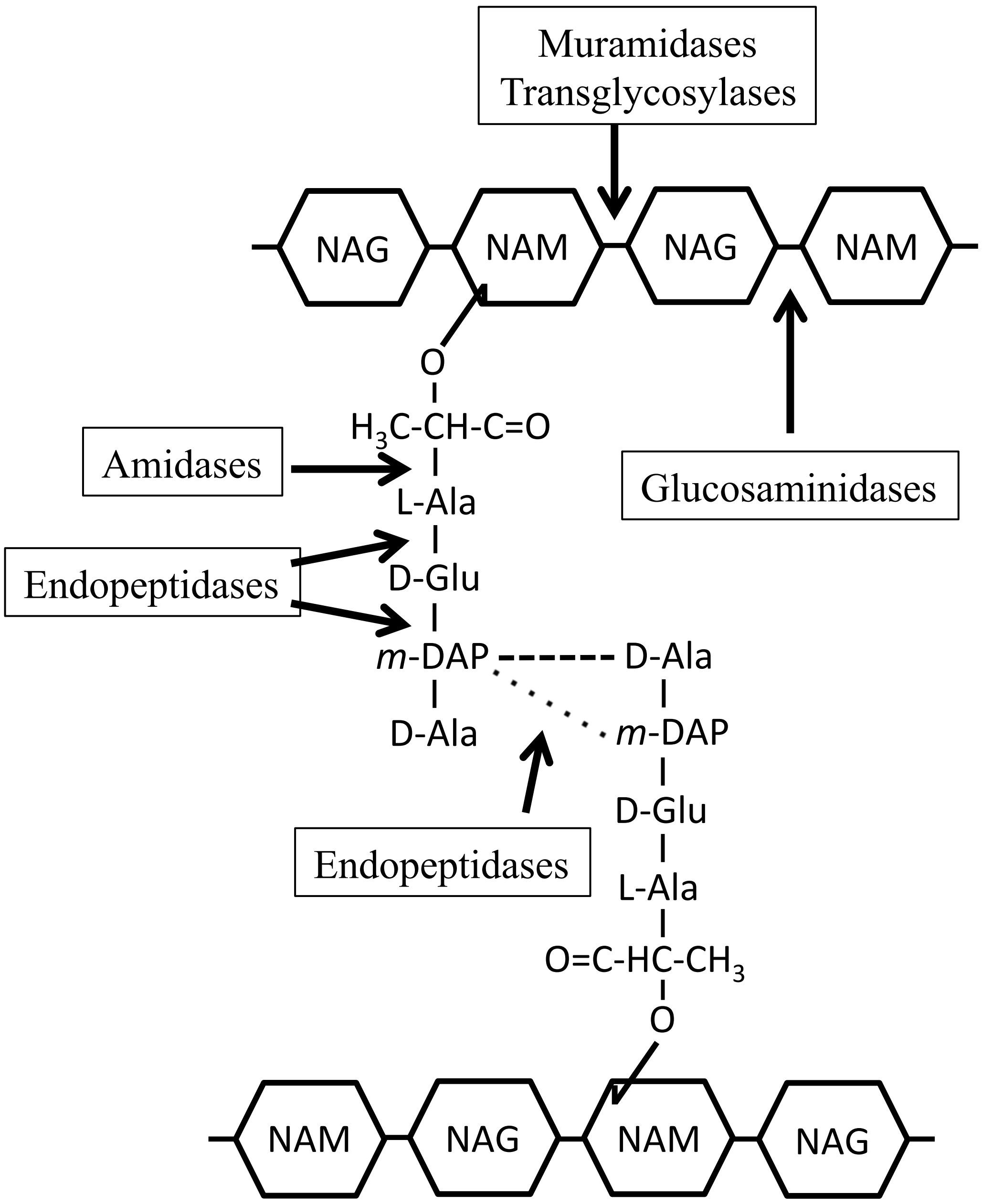


**Figure S1.** Schematic representation of the endolysins targets in the bacterial peptidoglycan. Proposed 4→3 interpeptide bridges between *m*-DAP and D-Ala but also 3→3 *m*-DAP to m-DAP bonds in the mycobacterial peptidoglycan are indicated by dashed lines. NAG, N-acetylglucosamine; NAM, N-acetylmuramic acid. In Pimentel M. Genetics of Phage Lysis. *Microbiol. Spectr.* **2014**, *2*, 1–13, doi:10.1128/microbiolspec.MGM2-0017-2013
