# Supplementary material for: Mycobacteriophage Lysis Enzymes: Targeting the Mycobacterial Cell Envelope
Source: Viruses. 2018 Aug 14;10(8):428. doi: 10.3390/v10080428 (PMC6116114; doi:10.3390/v10080428)
Supplement: Supplementary file 1 [file viruses-10-00428-s001.zip › viruses-331689-supplementary-final check/Figure S2.pdf]

CLUSTAL O(1.2.4) multiple sequence alignment

```
Dori_Gp41_Singleton           MSARLPLKLGSSD----------------------A-RGDDV---THWQRWA----RAYA  30
Sparky_Gp27_Singleton         MTARLPLKLGSSD----------------------A-RGDDV---THWQRWA----RAYA  30
Adjutor_Gp40_ClusterD         ----MELKVGSSD----------------------ANTDGEV---TRWQNWV----KRYA  27
Predator_Gp40_ClusterH        ------MGLPYKK----------------------GSNGPEI---KVWQDWA----YRYA  25
Nilo_Gp40_ClusterR            -----------------MRVNGVWVGWGV------GDHSAIDDTVRRAKAYMRAMYRSYA  37
Patience_Gp42_ClusterU        ----------------MKGPDGSYIGIGL------GDVSSEV---LKIKQFINRKFSRF-  34
Gaia_Gp34_ClusterX            ------------------------------------------------------------  0
Bricole_Gp38_ClusterM         ------------------MW----IGWQE------GMSGRPV---LAAKGELRAKFSYGK  29
TM4_Gp30_ClusterK             -----------------MAW----VGWQL------GMQGEQV---KVIQQKLIAKYQWVR  30
Courthouse_Gp48_ClusterJ      ----MPLRLGDRNES-VRQWRIKMNAWFGP--LYTRLHGPLP---MDTDEFGPRAKSWQE  50
Kumao_GP39_Singleton          -----------------MAW----DGWKE------GMAGPPV---LAAKRELKRKFSYAK  30
Cosmo_Gp52_ClusterV           -----------------MRVAGQWVGWGL------GDIDPKV---QDMKRFLKRKFSYAR  34
Ms6_LysB_ClusterF             -----------------MRIDGQYVGLGP------GDRSDEI---RKIKAFMRRKFSY-A  33
Corndog_Gp70_ClusterO         -----------------MRIGGEYVGLGL------GDASEEI---GRIRDFMRRKFRSYA  34
Bxz1_Gp238_ClusterC           -------------MR-VPPVTGEYVGLGL------GDSSEEI---RKIKEFMRKKFRSYA  37
MooMoo_Gp29_Singleton         -----------------MRIGGQYVGLGL------GDSSPEI---RNIKTFMRRKFASYA  34
D29_Gp12_ClusterA             ------------------------------------------------------------  0
LeBron-Gp26_ClusterL          --------------M-AKGLNGEWIGYGP------GDSGTQV---LAAREKLKAKFSY-G  35
MosMoris_Gp54_ClusterS        ----------MAKDPLTG-------AWIG---YGPGDVSPEV---QKVERRLLLAYP--K  35
32HC_Gp37_ClusterZ            -----------------M-------AWKQPQLTDPPMVSEEI---GKLNRRLLLAYA--A  31
BPs_Gp28_ClusterG             -----------------M-------AYQ-AP-RNVGDTHPLI---PAAKRKL----A--G  25
Giles_Gp32_ClusterQ           -----------------M-------AWKPTE-YQIGDRHPDI---ADAKERL-RRIA--A  29
Orion_Gp50_ClusterB           -------------MTQPT-------AWQPP--QNVGDVAVTV---AQAKAKL----K--V  29
Babsiella_Gp27_ClusterI       ---MTELRYGGAQPAEAM-------AWQKA--IREFAKSYAL---AADGGPL----K--I  39
Rubeelu_Gp27_ClusterN         ---MTELRIGSSGPL-VA-------AWQRA--MVDRFEAYAL---AADGGPL----R--V  38
Jebeks_Gp27_ClusterP          ---MTELRVGSSGPL-VA-------AWQQA--MVDRFEAYAL---AADGGPL----R--V  38
Cuke_Gp31_ClusterAC           ------------------------------------------------------------  0
Porky_Gp35_ClusterE           ------------------------------------------------------------  0
Bipper_Gp35_ClusterY          ------------------------------------------------------------  0


Dori_Gp41_Singleton           ESYAALMG-PVDGYYGNSDAEFTREMQRRLVAAGHAI------------AITGV-FDEAT  76
Sparky_Gp27_Singleton         ESYAALMG-PVDGYYGNSDAEFTREMQRRLVAAGHAV------------AITGV-FDEAT  76
Adjutor_Gp40_ClusterD         ASYSDIVG-PIDGYYGYSDADFTRELQRRLG-----L------------PITGV-FDDVT  68
Predator_Gp40_ClusterH        KSYADLIG-PKDAYFGNGEELFVKTMQGKLG-----V------------AQTGI-FGALE  66
Nilo_Gp40_ClusterP            GHLA------DTNEFDREMEAAVIEMQQRLVLQGRLE------L--G-TYVTGV-LDLPT  81
Patience_Gp42_ClusterU        -KLL------ETEIYDAAMEAAVRELQTIYKNNGT-L------T--D-PFIPGV-VNLAT  76
Gaia_Gp34_ClusterX            --------------------------------------------------MSGLWPEI-R  9
Bricole_Gp38_ClusterM         N-------LDLTEHFGPDLTEALKTFQRN---------------KGG-LRTDGV-LDYAT  65
TM4_Gp30_ClusterK             DRYP---RLTASGVYDVNTQAAIVEFQFRAGLPVT-----------------GI-ADYAT  69
```

```
Courthouse_Gp48_ClusterJ      ------------------------EYERR-------T------NQP----VDGEVSDNDL  69
Kumao_GP39_Singleton          H-------LVENTFFDHDLTVALMTYQVAKNIELAKR------GEPL-LRTDGV-LDWYT  75
Cosmo_Gp52_ClusterV           ESLD------DSEVYDETMMRVVMQMQANYGD----------------LDITGV-MNYAT  71
Ms6_LysB_ClusterF             ATLA------DTEFYDEAMTAVVAEMQSRYNTAGQLR------D--G-LYIPGI-INAET  77
Corndog_Gp70_ClusterO         GNLPDTRNALGLPLFDEPMTVAVTEMQGRYLQSGELR------D--G-LFIFGI-INAET  84
Bxz1_Gp238_ClusterC           GHLA------DTPHYDEQMTAVVAEMQGRYNQDGKLA------T--G-KYTPGI-INAET  81
MooMoo_Gp29_Singleton         GHLA------DTELYDEQMVAVVTEMQARYNASGQLA------S--D-KYTPGI-INAET  78
D29_Gp12_ClusterA             ------------------------------------------------------------  0
LeBron-Gp26_ClusterL          KHLK------LTTLYDAELLPVLVEFQLRTNVQRALT------GKQL-LRVDGI-LDYAT  81
MosMoris_Gp54_ClusterS        NSHAVEHGVQIDDVFTNGTAAALRDLTEFMNNDPREHERLAKLNVRLPMRSDGI-ADLNV  94
32HC_37_ClusterZ              NSRAVEAGVQLHDVFDAATDRALRNIQVHLAKTE----------DPKYNREPGV-LTYDC  80
BPs_Gp28_ClusterG             NSYGKAIGDDRSDVYTEAFGAALIQYGKNVHDLVLAG----RRPGPDVNV-EGI-FDWAI  79
Giles_Gp32_ClusterQ           KLVAGRLDNDNTDVFSPVFADVLAEWKTAVHRDVLSG----RRQPPDVDPTSTV-IDWAT  84
Orion_Gp50_ClusterB           FSYGAAFKTETSNVYTAEFGTALRTFQQRRNAEIHEG----KKPGPVMNT-DGV-LDWAT  83
Babsiella_Gp27_ClusterI       DGWI-----------GDDDAKVAAEYRSRR-------------RLPPPPP-GVV-VTHEE  73
Rubeelu_Gp27_ClusterN         DAYF-----------GYDDAAVQREYERRT-------------GQP--Q--DGV-VSEAD  69
Jebeks_Gp27_ClusterP          DAYF-----------GYDDAAVQREYERRT-------------QQW--I--DGV-VSEGD  69
Cuke_31_ClusterAC             ------------------------------------------------------------  0
Porky_Gp35_ClusterE           -----------------------------------------------------M-TTYGE  6
Bipper_Gp35_ClusterY          -----------------------------------------------------M-ATWDS  6


Dori_Gp41_Singleton           AALVGYAGA------PPAEERRPIWIYTAPGSGAPWN------------------VGPSF  112
Sparky_Gp27_Singleton         AAIVGYAGG---AKPPAATERRKIWIYTAPGSGAPWN------------------IGPSF  115
Adjutor_Gp40_ClusterD         ALHPKVNYRWKGQGAGQPAARRKIWFYSNPGSGANEF------------------VGPSF  110
Predator_Gp40_ClusterH        AGLTGFKAGS-VVISPPVIERRKIWFYSNPGSGANEF------------------VGPSF  107
Nilo_Gp40_ClusterP            QIAMG-------FKKAPN--PVRPIIITVEGHLSSMY------------------VGP--  112
Patience_Gp42_ClusterU        KYAIG-------YLKK-D--VILPIHFSVEGHMSDMW------------------IGP--  106
Gaia_Gp34_ClusterX            AMLNG-------EF-----YIPAGTIITANGTGVPDG------------------SGPGF  39
Bricole_Gp38_ClusterM         QKALG-------VLEA-----LKPWVFTVAGTGAGWD------------------AGY--  93
TM4_Gp30_ClusterK             QVRLG-------AVAPAPPPRQRIMVLTFSGTSADMW------------------TGY--  102
Courthouse_Gp48_ClusterJ      RA-------------LNVPVPTKVVIFTVAGTGARWD------------------QTY--  96
Kumao_GP39_Singleton          QK-------------VLGLLDRKVVIFTVSGTGAVWS------------------QGY--  102
Cosmo_Gp52_ClusterV           QVRSG-------YLKVEK--PPLPTLYTVHGTGVSMW------------------DGP--  102
Ms6_LysB_ClusterF             KYVMG-------YLSRPV-IDTRPVLFTVCGTGVPWW------------------VGP--  109
Corndog_Gp70_ClusterO         KYVMG-------YLERPPGPDTRPLFITVCGTGVPWW------------------VGP--  117
Bxz1_Gp238_ClusterC           KYVMN-------YLERPAGPDNRPVLFTVCGTGVPWW------------------VGP--  114
MooMoo_Gp29_Singleton         KYVMG-------YLQRPPGPDTRGVLLTVCGTGVPWW------------------VGP--  111
D29_Gp12_ClusterA             --------------------MSKPWLFTVHGTGQPDP------------------LGPGL  22
LeBron-Gp26_ClusterL          QVALG-------IVAPPA--VVKPLLFTVHGTGQPVP------------------DGPGL  114
MosMoris_Gp54_ClusterS        RRAIG-------AYID----P-----------PAAV--------------------QSKY  112
32HC_37_ClusterZ              KVRLG-------VIVP----A-----------PAAP--------------------DKRF  98
BPs_Gp28_ClusterG             QRQME-------LT------A----------PPAPPAPPRDRALAYVWRGTGGII-GQDL  115
```

```
Giles_Gp32_ClusterQ           KVQLG-------MIAR----A----------TPPAPAPPKARHLGIVFRGTGGII-GQDY  122
Orion_Gp50_ClusterB           KKQLG-------ILPE----Q---------TAPAPPPVPANRAAALVFRGTGGII-GQDY  122
Babsiella_Gp27_ClusterI       YAALV-------K--------------------TAPPPPKPRHLAIVFRGTGGVI-GQDY  105
Rubeelu_Gp27_ClusterN         LRALG-------L--------------------LDTPATKPRHLAIVFRGTGGAI-GQDY  101
Jebeks_Gp27_ClusterP          LRALG-------L--------------------LDTPATRPRHLAIVFRGTGGVI-GQDY  101
Cuke_31_ClusterAC             ---------------M----SDRIFFLWLPGTWEVDSLKAENPSATNFVGLGKWLVDDRF  41
Porky_Gp35_ClusterE           LKALR-------LGVK----YVRHTLFTVAGTWADMW--------------------SGY  35
Bipper_Gp35_ClusterY          ILDYL--------RGD----PCKVGYFGVPGTWSPWD--------------------AGY  34


Dori_Gp41_Singleton           ELGELCRKIL-----KLNHQPVG-YP--IGG----YLGLMGGDPALSYLDVIGAQAAEFA  160
Sparky_Gp27_Singleton         ELGELCRKIL-----KINHQPVG-YP--IGG----YLGLMGGDPALSYLDVIGAQAAEFA  163
Adjutor_Gp40_ClusterD         EVGEFCKNVL-----KINHQPVH-SA--IGG----YLGLMGGDPKFSYNDVIYDQYKSLE  158
Predator_Gp40_ClusterH        EVGEFCKNVL-----KINHQPVH-SA--IGG----YLGALGGDSKLSYNEVIFDQYKSIE  155
Nilo_Gp40_ClusterP            C-AYVASKL--ESEGVCWWQPIG-YD----------------NTSLPFRN--QTGISAVL  150
Patience_Gp42_ClusterU        A-AYVGEVL--RAENRALHFPTG-YD----------------NRALPFNN--KSGVEQLV  144
Gaia_Gp34_ClusterX            S-ADLARELDRRAEGMWVWRWIN-YP----------------AAVFPMRPSIGILRNNLK  81
Bricole_Gp38_ClusterM         P-AEVARAV----LDLYRWQGVN-YP----------------AKPFPMGSSVDAGIAELK  131
TM4_Gp30_ClusterK             P-ADVARALD---PSIFYWQPVC-YGPN--GI----------PAIFPMGSSAKSGEVEGL  145
Courthouse_Gp48_ClusterJ      P-YDLGRWQD---QNRVILQPIG-YP----------------AATFPMGPSVDKGEAELV  135
Kumao_GP39_Singleton          P-FDVAMRQD---QSKVIVQPIG-YP----------------AAVFPMEHSANEGERELV  141
Cosmo_Gp52_ClusterV           P-ADCARRL----LDKYRWQPVGNYP----------------ASAFPMWPSIQAGCLELN  141
Ms6_LysB_ClusterF             D-ADTARAV----EDQYLWQPIG-YP----------------AAPFPMGRSITAGITEAH  147
Corndog_Gp70_ClusterO         D-ADTARAC----EDKLFWQPIG-YP----------------AKNFPMGKSIAAGIDQCH  155
Bxz1_Gp238_ClusterC           D-AETARQV----EDLYRWQPVG-YP----------------AAPFPMGPSIEAGKAELR  152
MooMoo_Gp29_Singleton         D-ADTARAV----ENVYKWRPVG-YR----------------AAPFPMGASIAEGRAEAN  149
D29_Gp12_ClusterA             P-ADTARDV----LDIYRWQPIGNYP----------------AAAFPMWPSVEKGVAELI  61
LeBron-Gp26_ClusterL          P-ADTARNV----LDKWDWQPIGNYP----------------AEPFPMWPSILKGVAELR  153
MosMoris_Gp54_ClustersS       P-I---QGVF--------------HNTN---------AFLNPDPFHNFVQATNEGAAEAL  145
32HC_37_ClusterZ              V-Q---QGVG--------------FSTD---------AFLMGDPTHSYVDAVREGSAELL  131
BPs_Gp28_ClusterG             V-SLVCQGVA----DLVDEVNPP-WAATMGGIPVGV---AGGIGDPSMWSAVQATLAWTQ  166
Giles_Gp32_ClusterQ           V-SRVLQACA----DLVEEVHPA-FAATMGGIPVGT---AGGINDPSMAHAVALAFADAQ  173
Orion_Gp50_ClusterB           V-SQVCQQVG----PMVEEINPE-FPASMGGLPPGA------PNLPSARQAIDIGYRSGA  170
Babsiella_Gp27_ClusterI       V-SRVCQGAA----DLVEERNPE-WAASMGGLPPGA------PNSPSMNKAVQVAVASGA  153
Rubeelu_Gp27_ClusterN         V-SRVCQGAA----DLIEERNPE-FPASVGGLPPGA------PNSPSMNKAVQIGVAAGA  149
Jebeks_Gp27_ClusterP          V-SRVCQGAA----DLVEERNPE-WAASMGGLPPGA------PNSPSMNKAVQVAVASGA  149
Cuke_31_ClusterAC             P-DRLPLNIF---E-PIMLCPPD-YMASFGPIPAAGSSIFSAWNHLSYKESVLDAVEHSV  95
Porky_Gp35_ClusterE           P-ADVARLVD---EDLFRWQPVW-YPASFGPVGNP-------LG-RSYQESVQDGVKELI  82
Bipper_Gp35_ClusterY          Q-ADVGRLIN---SAWFYWQGVG-YVAAFGPVNGP-------ITNPSYAESVQEGVDETV  82
```

```
                                                              G-X-S-X-G
Dori_Gp41_Singleton          RLLRANPDVIEAMAARRRDRNARVDVEIWVSGYSQSADGFEDALEHLFGDGG--------  212
Sparky_Gp27_Singleton        RLLRINPDVIEAMAARRRDRNARVDVEIWVSGYSQSADGFEDALEHLFGDGG--------  215

Adjutor_Gp40_ClusterD        WLLDNNPDIKD------------PDVEFWFSGYSQKADGLEDALEILFGDGGFTIPKTGE  206
Predator_Gp40_ClusterH       WLLDNNPDIND------------PDLELWFSGYSQKADGLEDALEILFGDGGFVIPQTGE  203
Nilo_Gp40_ClusterP           NVLRQTVLLNG---NGQPTRPFPLGTPWGIEGFSQGAIIVNRILMLLRDTTD--------  199
Patience_Gp42_ClusterU       QRVGAAEFFI-----DGKVIKFPPGTPWTASAFSQGAMIWCDFYRQYLMPG---------  190
Gaia_Gp34_ClusterX           AMIRAT------------------PGRLVLSAYSQSAIAFAYVWRDDILNPN--------  115
Bricole_Gp38_ClusterM        KLLRERMD-------------RYPAARFVLIGYSQGAIVTSMVWQNFI---K--------  167
TM4_Gp30_ClusterK            RLLDEK---------------ARDFDYIVLIGYSQGALPASRLMRRIL---S--------  179
Courthouse_Gp48_ClusterJ     NQMRRHLD-------------ANPSLNFILVGYSQGAIVTSRVLRRMM---S--------  171
Kumao_GP39_Singleton         AQMRRHLD-------------ANPSYVFILIGYSQGAMVVSRVLRRMM---S--------  177
Cosmo_Gp52_ClusterV          RLIEST------------------PGKFTFAGYSQGAMVTSIVYKYDLLDPM--------  175
Ms6_LysB_ClusterF            NQANR-------------WRERIETHGTALAGYSQGAVVLSELWMNHIAPED--------  186
Corndog_Gp70_ClusterO        VQFNRADP-------GFMHRQRIERNGVVLGGYSQGAVVVSELWENNIKPAN--------  200
Bxz1_Gp238_ClusterC          AQINRMEP-------GFELRKQVERNGMVLAGYSQGAIVTSEVWEDDIRTSG--------  197
MooMoo_Gp29_Singleton        RII-------------VEERDRIEKYGLALAGYSQGAIVTSELWEYDIKPTS--------  188
D29_Gp12_ClusterA            LQIELKLD-------------ADPYADFAMAGYSQGAIVVGQVLKHHILPPT--------  100
LeBron-Gp26_ClusterL         VQIRKAFA-------------ANPNRKIGFAGYSQGAVVVSLVMKYDFMAEG--------  192
MosMoris_Gp54_ClusterS       RLYSTM-----------------PGRDIVVLGYSMGGVTAQKFLN---------------  173
32HC_37_ClusterZ             RLALPM-----------------VGVPKIGLAYSMGGDVLRAALE---------------  159
BPs_Gp28_ClusterG            TDFIARHK-------------VNPKIRVVIGGYSAGAIAAAMFRA---------------  198
Giles_Gp32_ClusterQ          RIFLERFR-------------ANPRIRVVIGGYSAGAVAAAMFRE---------------  205
Orion_Gp50_ClusterB          ----AWIK-------------ANPSRKFVLGGYSLGEIVVAKLLTALFSPG---------  204
Babsiella_Gp27_ClusterI      ----AEIR---------------SGRSFVLGGYSAGAIVASR-LRAMLEPG---------  184
Rubeelu_Gp27_ClusterN        ----AEIR---------------SGRSFVLGGYSAGAIVASR-LRAMLEPG---------  180
Jebeks_Gp27_ClusterP         ----AEIR---------------SGRSFVLGGYSAGAIVASR-LRAMLEPG---------  180
Cuke_31_ClusterAC            DVILKL----------------PTDRPIIIGGYSQGAEVAERL-KAEFLPG---------  129
Porky_Gp35_ClusterE          RLINA------------------TPGTFALVGYSQGAEVVSRV-LLEILFG---------  114
Bipper_Gp35_ClusterY         RLILA------------------RPGPIVLGGYSQGAEVVYWV-AREFLTG---------  114
                                                            .:*

Dori_Gp41_Singleton          -----EFELIRD--RLNGIIQFGNPSKDK-----------------T------GIAR-KT  241
Sparky_Gp27_Singleton        -----EFELIRD--RLNGVIQFGNPSKDK-----------------T------GIAR-KT  244
Adjutor_Gp40_ClusterD        TVGPGKYRHLRP--RINGTIQFGNPSKQPGPTRVG---NR---PPGS------GISR-KK  251
Predator_Gp40_ClusterH       KVGPGKYRHLRG--RINGVIQFGNPSKET-----------------T------GIAR-KQ  237
Nilo_Gp40_ClusterP           ----PMLQQRYA--DLTRGLTFGDPYREKDVVAE--WVTDPPKPGTQ------GISD--V  243
Patience_Gp42_ClusterU       ----KPLHWRLK--DLRAVICCGNPDREKGVCVD--WIPDKPGPDRQ------GIMDDEN  236
Gaia_Gp34_ClusterX           ----GELHHRLD--DIEAIILYGDPVRTPGIAYGNEL-GGVAPPGEVNGHVSGGIAG-PN  167
Bricole_Gp38_ClusterM        ----GS--DLEA--RIIGSITYGNPCRELHVANGNVA-EGIPVPEGR------G-IS-DF  210
TM4_Gp30_ClusterK            ----GDLQRFKS--KLIAGVTFGNPMREK----GHTF-PGGADPGGH------G-LD-PQ  220
```

```
Courthouse_Gp48_ClusterJ      ----GDLAHYYN--RCIAGVTFGNPMRER----GH-F-VGVNDPGGQ------G-LD-PK  211
Kumao_GP39_Singleton          ----GDLRQYFD--RCIAGVTFGNPLRER----GH-F-TGASDPGGQ------G-LD-PE  217
Cosmo_Gp52_ClusterV           ----GRLHHRLP--DFMGGVTWGNPMREMGKAWTDGV-GAVAGQNNG------G-IA-ED  220
Ms6_LysB_ClusterF             ----SGLRWMKP--HVRKAVTWGNPNRELGHVWADHGGSPMAPSNTQ------G-VS-SN  232
Corndog_Gp70_ClusterO         ----GSLHWAKD--YVVKAVTWGNPNREVGAVWPDYGGSPMASLTSQ------G-VS-ST  246
Bxz1_Gp238_ClusterC           ----PIVGWAKD--HVLKAVAWGNPNREQGKAYPDAG-APLAAADSA------G-IT-GR  242
MooMoo_Gp29_Singleton         ----GPLHWAKP--YVRKAVAWGNPMREIGKAWPDPG-APVSGMGRG------G-IT-QE  233
D29_Gp12_ClusterA             ----GRLHRFLH--RLKKVIFWGNPMRQKGFAHSDEWIHPVAAPDTL------G-IL-ED  146
LeBron-Gp26_ClusterL          ----GEFNALYKAGQVIAVVTWGNPMRERGVQHSDGT-KQVAPADTE------G-IL-ED  239
MosMoris_Gp54_ClustersS       ----RLPVEWRK--YVRALVTFGDPSMPAEGSLLG---ND---PG-E------GISK-AP  213
32HC_37_ClusterZ              ----KWPADRRG--EWSLFGVFGNPSKRPGPTLLG---ND---PGGQ------GISG-VW  200
BPs_Gp28_ClusterG             ----WLLENFPD--NYVCSFSFGDPTRPFGGGFFG---QP--APWGR------GIST-IS  240
Giles_Gp32_ClusterQ           ----WLLTNYPD--NYLCSFSFGDPTRPAGGAYFG---GV--AAPGR------GIST-WR  247
Orion_Gp50_ClusterB           ----GELAAFRD--NYVCSFHIGPPARPLGGAFYG---GT--AAPGV------GIAS-NR  246
Babsiella_Gp27_ClusterI       ----QPLAEYRE--NYVCGFALGNPSRPFGHTYYL---GA--IPNGR------GISD-FQ  226
Rubeelu_Gp27_ClusterN         ----QPLAAYRE--NYVCGFAFGNPCRPFGHTYYL---GA--IPNGR------GISD-FQ  222
Jebeks_Gp27_ClusterP          ----QPLAEYRE--NYVCGFALGNPSRPFGHTYYL---GA--IPNGR------GISD-FQ  222
Cuke_31_ClusterAC             ----GRLSSY----FLLAHYTFGNPGRPQGVTFPN---GN--TLPWG------GISN-LN  169
Porky_Gp35_ClusterE           -----SLRHRLK--DFIGGGCFGNPYRAKGVSYPG---SGLPTSGHG-------IAP-VN  156
Bipper_Gp35_ClusterY          ----GRLAHRRG--DLLLIVTFGNPCRAKGHGVNQ---AT--AHGWG-------ISR-K-  154
                                                    * *

                                                                   D
Dori_Gp41_Singleton           RPAWLL--------RLITNV----TTRG------DFYAEATD------------TIRPLF  271
Sparky_Gp27_Singleton         RPAWLL--------RLITNV----TTRG------DFYAEATD------------AIRPLF  274
Adjutor_Gp40_ClusterD         RPQWLT--------MMTWDIVTTSPGAP------DFYAACDD------------DIRPLF  285
Predator_Gp40_ClusterH        RPAWLR--------SLIRNV----TTKG------DFYAEAPD------------NIRPIF  267
Nilo_Gp40_ClusterP            RLTNTP--------SWWKVV----SRRG------DLYSENP-DN-------EVGLNRSAI  277
Patience_Gp42_ClusterU        RMVNTP--------WYWLEL----ARKG------DMYTDNESSG-------ERGLNKTAI  271
Gaia_Gp34_ClusterX            CLRPEECLHPVTGRRVILSV----ANPG------DLYASAPVGEVPWVEETEVGALETMI  217
Bricole_Gp38_ClusterM         RLRSTP--------SWWYDF----AHGANSRFGRDIYTDTP--------DDDAGEMMTAI  250
TM4_Gp30_ClusterK             CLVNTP--------DWWHDY----AAKG------DIYTVGSGSN-----DEKANADMTFI  257
Courthouse_Gp48_ClusterJ      PLVDTP--------SWWYDY----AARG------DIYSSGPGNN-----DRQAAEHMTSI  248
Kumao_GP39_Singleton          CLVDTP--------SWWHDY----AIPG------DIYTCGPGNY-----DLAALEHMRAI  254
Cosmo_Gp52_ClusterV           RLVGTP--------WNWRDY----AHKG------DLYTDCEFD------DEGE--YKRSV  254
Ms6_LysB_ClusterF             GMRNTP--------DWWRDY----AHQG------DLYACTEPG------DTQE--VRNAI  266
Corndog_Gp70_ClusterO         GMRDTP--------SWWRNY----AHAG------DLYAAAEPG------DSQQ--DKNAI  280
Bxz1_Gp238_ClusterC           LMTDTP--------DWWRNY----AHQG------DLYTATRPG------ESRE--DKVAI  276
MooMoo_Gp29_Singleton         LMVDTP--------EWWRNY----AHAG------DLYTDVADD------EAAE--NKRAI  267
D29_Gp12_ClusterA             RLENLEQY-----GFEVRDY----AHDG------DMYASIKED------DLHE--YEVAI  183
LeBron-Gp26_ClusterL          RLQGTP--------AMWREY----AHKG------DMYAACELT------GVQRGDNKRAI  275
MosMoris_Gp54_ClustersS       QPTWI--------RDRYWSY----SIDG------DWYPRARG-------------LLFLM  242
```

```
32HC_37_ClusterZ             YPEWT--------TGRLYDF----TLPG------DMYPNSVG-------------LLPQI 229
BPs_Gp28_ClusterG            YGDP--------TDYRHCWL----THEG------DMYAQIPG--------GVVGDIMDDV 274
Giles_Gp32_ClusterQ          YGDI--------RDYRHCWL----AAPG------DMYTSVPD--------NAVGDIMDTA 281
Orion_Gp50_ClusterB          LATDI----YAQLGPRACYL----CDPE------DMYGSIPVPV-----EGGTGDIMETV 287
Babsiella_Gp27_ClusterI      LPRSC----C---TWDWCEL----VHPD------DMYANVPL--------GDAGDIMTAI 261
Rubeelu_Gp27_ClusterN        LPRSC----C---TWDWCEL----VHPD------DMYANVPL--------GDAGDIMTAI 257
Jebeks_Gp27_ClusterP         LPRSC----C---TWDWCEL----VHPD------DMYANVPL--------GDAGDIMTAI 257
Cuke_31_ClusterAC            IP--T----PQG--TFYRSY----AFYD------DMYANANP--------K---SYLFEF 200
Porky_Gp35_ClusterE          LAPDI----LPA--EMWEEW----WNEG------DLYAQNLD--------GKSGEIITSF 192
Bipper_Gp35_ClusterY         -PPLT----ELL--PIWLDY----ALPG------DMYCCADD--------D---TYLAIG 186
                                                               * *

Dori_Gp41_Singleton          YEWFIRAETELPFVVYSAQI-ILPALLNLVAPFLG------------------GGLANPA 312
Sparky_Gp27_Singleton        YEWFIRAETELPFVVYSAQI-ILPALLNLVAPFLA------------------GGLASPL 315
Adjutor_Gp40_ClusterD        YEWFIKADTELPFVVYTAQI-IIPALLNLLAPFL----------------GGFGGVTSPL 328
Predator_Gp40_ClusterH       YQVIVDSESELPFFVRVLRI-AVPILIKWAATILPIFVP----L---AAAGGFGPMVQ-I 318
Nilo_Gp40_ClusterP           YKIAAENSW--------------------------------------------------- 286
Patience_Gp42_ClusterU       AKIITQNKW--------------------------------------------------- 280
Gaia_Gp34_ClusterX           FEAVMD------------------------------------------------------ 223
Bricole_Gp38_ClusterM        YRLVQDLKNI-------------------------------------------------- 260
TM4_Gp30_ClusterK            YQLVQGDILGMMFGTGNPLD------------IL-------------GLLGGLGG----- 287
Courthouse_Gp48_ClusterJ     YLAVMGKFIL-------------------------------------------------- 258
Kumao_GP39_Singleton         YLAVQGHFLT-------------------------------------------------- 264
Cosmo_Gp52_ClusterV          CKIVMG-HNV-------------------------------------------------- 263
Ms6_LysB_ClusterF            WQIVRD-LDL-------------------------------------------------- 275
Corndog_Gp70_ClusterO        WQIIRD-LNF-------------------------------------------------- 289
Bxz1_Gp238_ClusterC          WQIVRG-TNI-------------------------------------------------- 285
MooMoo_Gp29_Singleton        WAIVRG-TKV-------------------------------------------------- 276
D29_Gp12_ClusterA            GRIVMKASGF-------------------------------------------------- 193
LeBron-Gp26_ClusterL         CKIVMW-HEV-------------------------------------------------- 284
MosMoris_Gp54_ClustersS      YDILTRAALTMEFAV--YLFTQLPSRLFQELIGME--------------DSDDP----LA 282
32HC_37_ClusterZ             YQILVRMEASVEFAL--YLFNLLTSSFGPALLGLA--------------AGGLGPATAGF 273
BPs_Gp28_ClusterG            YEEVTRFAFRDILQVATRMVSAIPTI----AGKAGIPLP--------AVF---------- 312
Giles_Gp32_ClusterQ          YDIVTQVELSDFLGTAFGVARQIPII----MEEAGIGLP--------SVF---------- 319
Orion_Gp50_ClusterB          YDMVTTLALNDFLNTAAAMLPHILEI----AQDAGIFGL--------LGLGGVGPAT--- 332
Babsiella_Gp27_ClusterI      YQAVVDVELSDPLGTLRAIIRAIPTV----LAEAGVSVP--------L------------ 297
Rubeelu_Gp27_ClusterN        YQAVVEVELSDPLGTLRAIVASIPRV----LAEAGIKLP--------L------------ 293
Jebeks_Gp27_ClusterP         YQAVVDVELSDPLGTLRAIIRAIPTV----LAEAGVSVP--------L------------ 293
Cuke_31_ClusterAC            YDGLTDLQFHDPFKAVKDVVGVVTKSDLMILAGAQPTNPFWVVTHIPQFIDISTKAVN-- 258
Porky_Gp35_ClusterE          YDILTKLQFHDMLGLAVNMFKALSNDKGIIAQVMRVLAV-----PLPGVIDAG-RAAVYA 246
Bipper_Gp35_ClusterY         YAALTKLQLHDPWQLVQAMLALIQSDEF-ADALAELLDP-----LFPGLATTLGELTGMD 240
```

```
Dori_Gp41_Singleton           ALPILAAVTGAGSGLLGSVIGGVL----GAK---------------------------D-  340
Sparky_Gp27_Singleton         AAPVLAGVTGAGGGLLNTVIGGVL----GAK---------------------------D-  343
Adjutor_Gp40_ClusterD         AGGILASATGLPMNLLHGLLSGVA----AAD---------------------------N-  356
Predator_Gp40_ClusterH        ALSTLSGLQGLGSNPLFGSLMGQA----GND-------------------------GNA-  348
Nilo_Gp40_ClusterP            ------------------------------------------------------AGGPA-  291
Patience_Gp42_ClusterU        ------------------------------------------------------SGGPA-  285
Gaia_Gp34_ClusterX            ------------------------------------------------------FNGRDF  229
Bricole_Gp38_ClusterM         ------------------------------------------------------IVGTD-  265
TM4_Gp30_ClusterK             ---------GLLGGLGGGLLGGGKGGLQLPSGLVLPGVQGGAL-----------TDHQR-  326
Courthouse_Gp48_ClusterJ      --------------------------------------------------------GND-  261
Kumao_GP39_Singleton          --------------------------------------------------------GRD-  267
Cosmo_Gp52_ClusterV           ------------------------------------------------------FGGPD-  268
Ms6_LysB_ClusterF             ------------------------------------------------------FTGPD-  280
Corndog_Gp70_ClusterO         ------------------------------------------------------FTGTD-  294
Bxz1_Gp238_ClusterC           ------------------------------------------------------LSGPD-  290
MooMoo_Gp29_Singleton         ------------------------------------------------------FSGPD-  281
D29_Gp12_ClusterA             ------------------------------------------------------IGGRD-  198
LeBron-Gp26_ClusterL          ------------------------------------------------------LKGEN-  289
MosMoris_Gp54_ClusterS        GALAPL------------------------GDLL---------MAGRGIL----------  299
32HC_37_ClusterZ              GALSSI------------------RSMVTIGGLGM------APATSAGDV----------  299
BPs_Gp28_ClusterG             ------------------------------------GALAGGPAGLTTFAIPLLL-----  331
Giles_Gp32_ClusterQ           ------------------------------------KALAGGPAGLAGLGVPLIM-----  338
Orion_Gp50_ClusterB           ----------GGGGLLGGLLGGGLGGLLGGG-MANPAALLANPLAAIPLLLPLFT-----  376
Babsiella_Gp27_ClusterI       --------------------------------LAHAGLSAGNPVEMAGVALPVLT-----  320
Rubeelu_Gp27_ClusterN         --------------------------------FAPPAASA-EPAAAAAMLLPALT-----  315
Jebeks_Gp27_ClusterP          --------------------------------LAHAGLSAGNPVEMAGVALPVLT-----  316
Cuke_31_ClusterAC             ------------------------------------------------------------  258
Porky_Gp35_ClusterE           GTFAVQGTRPHITLAETGRVARAVWHLNRIGAKTLARAS---------------------  285
Bipper_Gp35_ClusterY          GAALLANRKPVGGGVLGGLMSGQLLTTTPAGGNLLGGLIGAGTGVLGGLLDGVIPGGLP-  299

                                                                            H
Dori_Gp41_Singleton           -KPNPELIELLSVR---GVLTNLGELIAL-------LAALP-GLQAHGEYHLP-KPEFNG  387
Sparky_Gp27_Singleton         -KPNPELIELLSVR---GVLTNIPQLVAL-------LAALP-GLQSHGEYHLP-KPEFNG  390
Adjutor_Gp40_ClusterD         -APNPKLIELLSVR---GVLTNIPQLIKL-------LTNIS-GVQTHGEYHLP-KPEFNG  403
Predator_Gp40_ClusterH        -EEDRKMIELLSPT---GVLTNIPGLIQL-------IAALP-GLQAHGEYHLP-KAEFNG  395
Nilo_Gp40_ClusterP            -GLLQRIGDFLMD-----PLDGTIDIGLAIIGGVMF-MGN---MEPHGGYDLN--PC---  336
Patience_Gp42_ClusterU        -GLLARVTDLLVN-----PIDDVIPITLALYDAIRFGAGG---IRAHGGYDME--PA---  331
Gaia_Gp34_ClusterX            LAFAKEIAELFIK-----PLSQVLPLVQAIWNGLTFLGQGS--SAPHWTYNVM--PA---  277
Bricole_Gp38_ClusterM         -SLIEQVGEMFQR-----PITEVGAAMWAIFLGGQFVATRPYPTMPHINYDIN--PAVAI  317
TM4_Gp30_ClusterK             -GLVEAVLALLAN-----PFAEVPAAVKAIVSGVGFIATNP-PTAPHIEYHIR--EAAPG  377
Courthouse_Gp48_ClusterJ      -ALVSQVIELFTN-----PFAEVPAVVKAIASGIGFVTSNP-PTAAHIEYHIR--ECVPG  312
Kumao_GP39_Singleton          -NLGEQVLEVLMN-----PFAEVPAVVKAIVSGLGFVTANP-PTAPHIEYHIR--ECFPG  318
```

```
Cosmo_Gp52_ClusterV          -SILRQVIELGLD-----PFGEAIPMIKAISDAGMFFINR---TTPHINYNVG--PA---  314
Ms6_LysB_ClusterF            -SLLAQVIELAQA-----PLPETIAITRAILDAGMFFAKR---TGPHVDYNPQ--PA---  326
Corndog_Gp70_ClusterO        -SLLAQAIELSQM-----PIPRTIAAFKALIDAGMFFAKG---TGPHVDYGIG--PA---  340
Bxz1_Gp238_ClusterC          -SLLRQFLEIAEA-----PVPNAIAAFQAFMDAGLFFVKG---TRPHTNYHIG--AA---  336
MooMoo_Gp29_Singleton        -SILAQVLEVMGIRQDAGMVMEVMAIFKAIMDAGLFFLKG---TGPHVNYNIQ--PA---  332
D29_Gp12_ClusterA            -SVVAQLIELGQR-----PITEGIALAGAIIDALTFFARSRMGDKWPHLYNRY--PA---  247
LeBron-Gp26_ClusterL         -NILAQIAELLQQ-----PVLHIIPLFQSIVDAGMFFTAG---VDGPHNYAIQ--PA---  335
MosMoris_Gp54_ClusterS       --------------NPAQMFAILPDLFNLLFDAIKFV-----ATNAHGKYGDPGYALWGG  340
32HC_37_ClusterZ             --------------NLMAMITNIPAIIQSIAAALKFV-----QTNAHYHYHDQPQPYWRG  340
BPs_Gp28_ClusterG            -SSIRGFIPG----GNADELTGTAAAKAATIGLQFLFA---GTAPHIRYHID--EAWPG  381
Giles_Gp32_ClusterQ          -GLLGGLIGG----QK--NPTGVAAAAQAAVIALQFVTSNP-PTAAHIQYEFR--EVWPG  388
Orion_Gp50_ClusterB          -SALPGLIAGVGGPGTGGALTGPAAAAQAAILGMKFLFA---GTRPHIEYHIR--EVWPG  430
Babsiella_Gp27_ClusterI      -STLAGLIGG----AAGGPLTGPAAAVQAAIIAIRFAAS---GTAPHINYHAW--EVWPG  370
Rubeelu_Gp27_ClusterN        -ATLPGLTGA----D-PARPSGPAAAVQAAIIALRFAAS---GTAPHINYHAW--EVWPG  364
Jebeks_Gp27_ClusterP         -STLGGLVGG----AVGGQLTGPAAAVQAAIVALKFAVA---GTAPHINYHAW--EVWPG  366
Cuke_31_ClusterAC            ----------------------------SLDALARFA-----SSGAHGHYGDW--EIIPG  283
Porky_Gp35_ClusterE          ------------------------------------------------------------  285
Bipper_Gp35_ClusterY         -PIVGGLLGGGS--GPGGTPTGMYKLGKTFAALLNFA-----TTNDHGHYHDT--PAFAG  349


Dori_Gp41_Singleton          -RTGIQVAYDVVASFRR---PPIASP-------------------     409
Sparky_Gp27_Singleton        -RTGIQVGYDVVAAFRR----------------------------     406
Adjutor_Gp40_ClusterD        -RSGIQVGCDIVAGFRR----------------------------     419
Predator_Gp40_ClusterH       -RTGIQVGCDIVAAFRR----------------------------     411
Nilo_Gp40_ClusterP           --------VEYMRGVGTRRVA------------------------     349
Patience_Gp42_ClusterU       --------VQFCRERLAA---------------------------     341
Gaia_Gp34_ClusterX           --------ADYLIELGANIRERGVA--------------------     294
Bricole_Gp38_ClusterM        -LRRIAA--------------------------------------     323
TM4_Gp30_ClusterK            -VTYFQHAIDYLRQVGASVAARAA---------------------     400
Courthouse_Gp48_ClusterJ     -VTYFDHAMGYVRQVISANKRIA----------------------     334
Kumao_GP39_Singleton         -VTHFEHAVDYVRRAVSAGMRIE----------------------     340
Cosmo_Gp52_ClusterV          --------VDFLAGL------------------------------     321
Ms6_LysB_ClusterF            --------IDYLRT-------------------------------     332
Corndog_Gp70_ClusterO        --------IEYLRS-------------------------------     346
Bxz1_Gp238_ClusterC          --------VDYLRS-------------------------------     342
MooMoo_Gp29_Singleton        --------IEYLRAT------------------------------     339
D29_Gp12_ClusterA            --------VEFLRQI------------------------------     254
LeBron-Gp26_ClusterL         --------IDYLRSVV-----------------------------     343
MosMoris_Gp54_ClusterS       -MTAVDHAAATIRERVPNGATLFLLPGTWSMWNQLFPFDTAVRLQ     384
32HC_37_ClusterZ             -LTGVDCAAQVISESVE-RATVFTVPGTVSHWNDGPPAWTAWKLP     383
BPs_Gp28_ClusterG            GPTFLDLARQHVRDWTSR--PAA----------------------     402
Giles_Gp32_ClusterQ          -QTYLGLAIQHVRDWAGR--TPAVTA-------------------     411
Orion_Gp50_ClusterB          -QTYIGLAVQHVRDWVGR--ELPA---------------------     451
```

```
Babsiella_Gp27_ClusterI      -QTYLGLAVQHVRDWAQR--VPVRA--------------------    392
Rubeelu_Gp27_ClusterN        -QTYLGLAIQHVRDWASR--TPVRN--------------------    386
Jebeks_Gp27_ClusterP         -QTYLGLAIQHVRDWASR--TPVRA--------------------    388
Cuke_31_ClusterAC            -FTPIFHCIRSAKYAAKG--IGYAVPGI-----------------    308
Porky_Gp35_ClusterE          ---------------------------------------------    285
Bipper_Gp35_ClusterY         -TNAVAHAVGEVNRLAA----------------------------    365
```

Figure S2. Alignment of LysB proteins. The conserved pentapeptide (G/A-X-S-X-G) is colored pink. The conserved amino acids candidates to be part of the catalytic triad, Ser, Asp and His are highlighted in pink, light blue or yellow backgrounds respectively. The already identified catalytic triad of LysB D29 Ser82, Asp166 and His240 are colored red. The dashed blue lines represent the absent N-terminus region. Numbers refer to the amino acid positions. A representative of each cluster, for which a *lysB* gene was identified, was selected from the Actinobacteriophage database (www.phagesdb.or). Accession numbers are indicated in Table S1.
