# Supplementary material for: Mycobacteriophage Lysis Enzymes: Targeting the Mycobacterial Cell Envelope
Source: Viruses. 2018 Aug 14;10(8):428. doi: 10.3390/v10080428 (PMC6116114; doi:10.3390/v10080428)
Supplement: Supplementary file 1 [file viruses-10-00428-s001.zip › viruses-331689-supplementary-final check/Table S1.pdf]

Table S1: Coordinates of LysB coding genes

Phage Name	Cluster	LysB coding gene	Position		Number of AA	Pham	Extended N-terminus	LysB Acession Number
			start	end				
D29	A	gp12	6606	7370	254	45275	-	AAC18452
Orion	B	gp50	41837	43192	451	609	+	ABD58366
Bxz1	C	gp238	145870	146898	342	45104	+	AAN16870
Adjutor	D	gp40	32695	33954	419	4477	+	ACD49625
Porky	E	gp35	31695	32552	285	45302	-	ACF33852
Ms6*	F	gp3	2371	3369	332	45104	+	AAG48319
BPs	G	gp28	25201	26409	402	609	+	ACB58187
Predator	H	gp40	33027	34262	411	4477	+	ACF05137
Babsiella	I	gp27	24108	25286	392	609	+	AER48404
Courthouse	J	gp48	39086	40090	334	45262	+	AER47899
TM4	K	gp30	24984	26186	400	6899	+	AAD17597
LeBron	L	gp26	25573	26604	343	45104	+	ADL70993
Bricole	M	gp38	31780	32751	323	45104	+	ALF00566
Rubeelu	N	gp27	22757	23917	386	609	+	AXC38538
Corndog	O	gp70	44462	45502	346	45104	+	AAN02002
Jebeks	P	gp27	24037	25203	388	609	+	AVD98065
Giles	Q	gp32	28339	29574	411	609	+	ABW88427
Nilo	R	gp40	35349	36398	349	45104	+	AVO21438
MosMoris	S	gp29	31775	32929	384	928	+	AHY84128
Patience	U	gp42	35792	36817	341	45256	+	AEL97951
Cosmo	V	gp52	38381	39346	321	45283	+	AJD82124
Gaia	X	gp34	30705	31589	294	8237	-	AID58854
Bipper_	Y	gp35	30012	31109	365	18051	-	AMQ66971
32HC_	Z	gp37	28787	29938	383	928	+	AHJ86315
Cuke	AC	gp31	27339	28265	308	24285	-	AVD99649
Dori_	Singleton	gp41	36640	37869	409	4477	+	AER47691
Kumao	Singleton	gp39	34279	35301	340	45104	+	ATN94002
MooMoo_	Singleton	gp54	26730	27749	339	45104	+	AVO21635
Sparky	Singleton	gp27	25276	26496	406	4477	+	AII28175

*Ms6 coordinates regards to the lytic cassette since the complete genome sequence is not yet available
